# SbD4Skin by EosCloud: Integrating multi-view molecular representation for predicting skin sensitization, irritation, and acute dermal toxicity

**DOI:** 10.1016/j.csbj.2025.08.001

**Published:** 2025-08-06

**Authors:** Nikoletta-Maria Koutroumpa, Dimitra-Danai Varsou, Panagiotis D. Kolokathis, Maria Antoniou, Konstantinos D. Papavasileiou, Eleni Papadopoulou, Anastasios G. Papadiamantis, Andreas Tsoumanis, Georgia Melagraki, Milica Velimirovic, Antreas Afantitis

**Affiliations:** a Entelos Institute, Nicosia 2102, Cyprus; b School of Chemical Engineering, National Technical University of Athens, Athens 15780, Greece; c NovaMechanics MIKE, Piraeus 18545, Greece; d Division of Physical Sciences & Applications, Hellenic Military Academy, Vari 16673, Greece; e Flemish Institute for Technological Research (VITO), Boeretang 200, Mol 2400, Belgium

**Keywords:** Skin sensitization, skin irritation, acute dermal toxicity, explainable predictions, machine learning, molecular representations, SbD4Skin, EosCloud

## Abstract

Assessing chemical toxicity is essential for understanding potential risks to human health. However, ethical, financial, and scientific challenges have driven the demand for non-animal testing methods. This study introduces a computational framework that leverages diverse molecular representations, including MACCS keys, Morgan fingerprints, and Mordred descriptors, to predict skin sensitization, irritation/corrosion, and acute dermal toxicity. Different molecular representations for skin toxicity-related endpoints were first evaluated using three machine learning algorithms (Random Forest, Support Vector Machine, and k-Nearest Neighbors), then combined into a unified input space for training a fully connected neural network (FCNN). Comparative analyses indicate that this multi-view FCNN model offers superior or comparable predictive performance relative to single-representation models, achieving area under the curve (AUC) values of up to 0.91 for irritation/corrosion, 0.88 for sensitization, and 0.82 for acute dermal toxicity on test sets. Additional validation on known toxicants further confirms the framework’s robustness, correctly identifying 0.86 of skin sensitizers, 0.89 of irritants, and 0.86 of dermally toxic compounds. Shapley Additive exPlanation (SHAP) analyses highlight the most influential molecular features, providing mechanistic insights and enhancing model transparency. To promote broader adoption and reduce reliance on animal testing, the developed models are freely available through the SbD4Skin (Safe by Design for Skin) web platform (https://eoscloud.entelos.eu/ssbd4chem/sbd4skin/), offering a user-friendly tool for chemical risk assessment and regulatory decision-making. The dataset and model developed in this study have been FAIRified and made available in machine-actionable and modelling-ready formats, supporting transparency, reuse, and regulatory acceptance.

## Graphical Abstract

**Figure:**
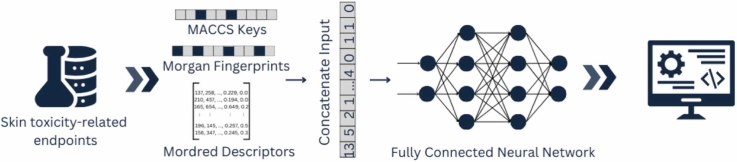

## 1 Introduction

Toxicity assessment plays a crucial role in evaluating the potential risk of chemicals to human health and the environment [1]. Traditionally, regulatory frameworks have relied on *in vivo* testing for hazard categorization, which provides valuable data on toxicological endpoints [2]. However, several factors are driving international regulatory agencies to replace animal use, including ethical concerns, financial costs, and scientific limitations regarding the relevance of animal models to human responses [3]. With the advent of the Registration, Evaluation, Authorization and Restriction of Chemicals (REACH), alongside EU Commission and U.S. Food and Drug Administration regulations, there is a growing preference for alternative, non-animal testing methods for toxicity evaluation [4]. These initiatives align with the principles of New Approach Methodologies (NAMs), which promote *ex vivo*, *in vitro*, *in chemico* and *in silico* strategies [5]. NAMs aim to Reduce, Refine, and Replace (3Rs) animal testing for hazard identification, thereby enhancing animal welfare globally [6].

Among various toxicity endpoints, skin sensitization, irritation/corrosion, and dermal toxicity are of particular regulatory and industrial importance, as they are essential for consumer safety in pharmaceuticals and cosmetic products [7]. Traditional *in vivo* methods have been widely used, such as the mouse-based local lymph node assay (LLNA) for the identification of skin sensitization hazards [8], and the Draize rabbit skin test [9] for skin irritation/corrosion test. However, these methods are being replaced by alternative approaches, such as *in vitro* methods using reconstructed human epidermis (RhE) [10], as well as *in silico* approaches, which leverage computational techniques for toxicity prediction. The cosmetics industry, in particular, has strong motivation to adopt non-animal approaches to generate toxicological data due to the full implementation in 2013 of the EU Cosmetics Regulation No. 1223/2009 [11], which prohibits the testing of cosmetic ingredients on animals. This regulatory shift has catalyzed substantial innovation in *in silico* toxicology, which now serves as a fundamental tool for chemical safety assessment. These computational methods offer a cost-effective, rapid, and ethically sound alternative to traditional testing paradigms, enabling the high-throughput evaluation of large chemical libraries [12]. As a result, the dermo-cosmetic field has increasingly incorporated computational methods to identify compounds with potential adverse effects, prioritize candidates for follow-up *in vitro* testing, and support data-driven regulatory submissions [13].

Recent advances in computational toxicology highlight the versatility and scientific value of these approaches. For instance, Limluan *et al*. developed a quantum mechanical model using density functional theory (DFT) to estimate the skin sensitization potential of acyl-containing chemicals, demonstrating a strong correlation between reaction barriers and sensitization potency [14]. With the advancement of NAMs, defined approaches (DAs) integrate *in silico, in chemico*, and *in vitro* methods [15]. Ankli *et al*. compared the results of different DAs for skin sensitization assessment [16], [17]. One of the key advancements reported by Mohoric *et al*. was the integration of kDPRA data, which measures covalent binding to skin peptides and proteins. This integration enhanced the prediction of LLNA potency classes, achieving accuracy rates between 0.63 and 0.88 [18].

Computational approaches, including read-across and quantitative structure–activity/toxicity relationship (QSAR/QSTR) modeling, have been introduced to facilitate risk assessment of new chemicals [19, 20]. Read-across is a data-driven technique for predicting the toxicity of untested substances based on structurally similar chemicals with known toxicological profiles [21]. QSAR-type models, on the other hand, rely on mathematical and statistical methods to establish correlations between chemical structure attributes and specific adverse effects. While numerous QSAR-type models are currently available for diverse endpoints, they must be validated according to Organization for Economic Cooperation and Development (OECD) Guidance to meet regulatory requirements [22].

Recent progress in machine learning (ML) and deep learning (DL) has further enhanced the predictive accuracy of QSAR-type models by capturing complex, non-linear relationships between chemical structures and biological activity [23, 24]. Consequently, various research groups have developed reliable QSAR-type models to forecast skin toxicity endpoints, leveraging different ML algorithms and molecular representations. For example, Pred-Skin is a two-stage trained system in which individual QSAR models were first trained on five different skin sensitization data sets. The outputs of these models were then used as inputs to train a Bayesian model on the human data, resulting in an integrated decision model for predicting human skin sensitization effects. Pred-Skin achieved a correct classification rate of 0.89 on their test set [25]. Later, Wang *et al*. applied the Dempster-Shafer theory (DST) to merge multiple QSAR models into an evidence-based framework for skin sensitization prediction [26]. Similarly, Tieghi *et al*. introduced HuSSPred, one of the first *in silico* tools trained on human data from the Human Predictive Patch Test (HPPT), specifically designed to predict human skin sensitization effects of chemical agents [27]. HuSSPred demonstrated strong predictive performance with correct classification rate ranging from 0.55 to 0.88. DL frameworks employing recurrent neural network architectures (RNNs), SMILES tokens, fingerprints and descriptors were developed by Duy and Srisongkram for skin sensitization [28] and for skin irritation [29], achieving an accuracy of 0.87 and 0.80, respectively.

For predicting skin irritation and corrosion, AttentiveSkin was developed using an interpretable deep learning framework (Attentive FP) trained on a large, curated dataset comprising compounds from multiple regulatory sources. The model delivered robust performance, with AUCs of 0.97 and 0.85 for corrosion and irritation, respectively [30]. Kang *et al*. investigated the prediction of skin irritation and corrosion in liquid chemicals using 34 physicochemical descriptors; among several ML algorithms, XGBoost demonstrated the highest accuracy (0.73) [31]. For acute dermal toxicity, Lou *et al.* utilized molecular fingerprints and graph-based descriptors to train ML models on rat and rabbit data, achieving external AUCs of 0.63 and 0.76, respectively [32]. The STopTox project compiled curated datasets for six toxicity endpoints, including skin sensitization, skin irritation and corrosion, eye irritation and corrosion, acute dermal toxicity, acute inhalation toxicity and acute oral toxicity, and developed QSAR models in accordance with the OECD guidelines [33]. Using Morgan fingerprints, molecular access system keys (MACCS keys) and Mordred descriptors with Random Forest (RF) classifier, the consensus models achieved external correct classification rates of 0.70–0.77. Chushak *et al.* [34] developed ten classification model for the same six toxicity endpoints addressed in STopTox, combining ML and DL algorithms. Model performance was evaluated using different applicability domain definitions, such as similarity-based and distance-to-model approaches. Notably, the use of a distance-to-model approach yielded prediction accuracies ranging from 0.75 to 0.86 across different toxicity endpoints.

QSAR models are typically based on fixed molecular representations, and they often fail to fully capture both local and global information in molecular structures. A study that addressed this limitation was conducted by Fuadah *et al.* [35]. They curated the data from STopTox, RespiraTox [36] and DMFGAM [37] and presented a hybrid ML approach that integrates best performing algorithms from previous studies, including XGBoost, Support Vector Machines (SVM) and RF with different featurization methods, presenting a consensus model with AUC scores ranging from 0.78 to 0.90.

In the present study, we investigate the comparative performance of multiple molecular representations including MACCS keys, Morgan fingerprints, and Mordred descriptors, across three machine learning algorithms, namely RF, SVM and k-Nearest Neighbors (kNN). We further integrate these representations into a combined input space to train a fully connected neural network (FCNN). All models are optimized via 5-fold cross validation to determine the optimal hyperparameters. The results demonstrate that incorporating multiple chemical representations improves predictive accuracy, yielding more robust models. We validated our models on both an internal test set derived from the original dataset and an external validation set consisting of known toxicants from the literature. To promote transparency, reusability, and regulatory compliance, the study adopts the FAIR (Findable, Accessible, Interoperable, Reusable) data principles [38] and the respective GO FAIR Foundation interpretations [39] for data and metadata. For this purpose, the developed models are properly documented through the QSAR Model Reporting Format (QMRF) documentation standard [40] and the MODA [41], which provides a standardized structure for documenting data-driven models and their predictions. Furthermore, the models are made available through an intuitive web-service hosted in the EosCloud (https://eoscloud.entelos.eu/ssbd4chem/sbd4skin/) that can be used for virtual screening applications. Finally, all data used to develop the models are available through the ChemPharos database [42], a database for advanced chemical data management.

## 2 Materials and methods

### 2.1 Data collection and preprocessing

The datasets for this study were obtained from Borba *et al*., where the detailed curation process is presented [33]. This process included the removal of inorganics and mixtures, cleaning of salts, normalization of chemotypes, the removal of duplicates, and manual inspection of the final dataset. Each endpoint is classified according to the Globally Harmonized System of Classification and Labeling of Chemicals (GHS) hazard classes. To convert the data into the binary toxicity classes, we followed the classification scheme as described in Borba *et al*. [33] in which: for skin sensitization, GHS category 1 was considered “sensitizer”, for skin irritation, categories 1–3 were considered as “irritant or corrosive” and for acute dermal toxicity, GHS categories 1–4 were considered as “toxic”, while category 5 was considered as “not-classified”. This binarization strategy enables the development of screening-level predictive models by simplifying complex classification tasks. However, we acknowledge that such simplification may limit the models’ use in regulatory settings where finer subcategory distinctions are needed. Nonetheless, the binary categorization is fit for purpose in the context of early-stage safety screening.

Skin sensitization data were collected from the National Toxicology Program Interagency Center for the Evaluation of Alternative Toxicological Methods on behalf of ICCVAM [43] and the REACH Study Results Database [44]. After curation, 1000 unique compounds remained, with 481 classified as sensitizers and 519 as non-sensitizers. Skin irritation/corrosion data were retrieved from the REACH Study Results Database [44]. The curated dataset contained 1012 compounds, of which 317 were irritants and 695 were non-irritants. Due to the imbalance in these two classes, an under-sampling procedure was carried out to balance the dataset while minimizing information loss from the majority class. Specifically, the compounds were grouped into 317 clusters through k-Means clustering (scikit-learn implementation) based on Morgan fingerprints [45], also known as extended-connectivity fingerprint ECFP4 [46]. One compound per cluster was selected until the number of majority-class compounds matched that of the minority class. Consequently, the balanced dataset consisted of 634 compounds (317 irritants and 317 non-irritants). Acute dermal toxicity was retrieved from the REACH Study Results Database [44], the ToxValDB [47] and from the literature [48]. The original collection, stored in STopTox [33], featured 2616 compounds, comprising 382 dermally toxic chemicals and 2234 non-dermally toxic chemicals. The under-sampling strategy mentioned above was applied, resulting in a balanced dataset of 764 compounds, of which 382 were dermally toxic and 382 were non-dermally toxic. To further confirm that under-sampling did not distort the chemical space, the selected and removed majority-class compounds were visualized via t-distributed stochastic neighbor embedding (t-SNE), a dimensionality reduction method proficient at capturing non-linear structures (Figures S1 and S2 in Supplementary Material). The t-SNE plots indicate that the overall distributions are maintained, with both retained and removed compounds exhibiting similar coverage. Thus, the under-sampling procedure did not lead to substantial information loss.

### 2.2 Feature engineering

In this study, multiple molecular representations were examined including Morgan fingerprints [46], MACCS keys [49], Mordred descriptors [50] and an integrated combination of these representations, because each provides unique insights into chemical structure and properties. Adopting various representations allows for a more comprehensive view of molecular features, potentially leading to improved predictive accuracy. Morgan fingerprints [46] rely on circular atomic neighborhoods to generate variable-length hashed integer identifiers, each corresponding to a unique substructure within a molecule. This approach captures local connectivity and substructural patterns, making it particularly adept at encoding structural motifs that may be relevant to toxicity endpoints. MACCS keys [49] detect commonly occurring chemical functionalities, such as aromatic rings or ring structures, based on a predefined set of binary questions about the presence or absence of specific substructures. While MACCS keys are shorter than many other fingerprint types, they can still highlight critical functional groups often associated with biological activity or toxicity. Mordred descriptors [50] constitute a set of more than 1800 two- and three-dimensional descriptors that capture geometric, electronic, topological, and hybrid characteristics. In this study, only the two-dimensional Mordred descriptors were utilized to ensure consistency with other molecular representations. Unlike fingerprints, which are typically binary or hash-based, Mordred descriptors provide continuous values describing various molecular attributes (e.g., molecular weight, electronegativity, shape factors), thereby delivering a richer and more granular representation of each compound’s physicochemical properties.

By integrating these representations, the study aimed to exploit the strengths of each method, since Morgan fingerprints can emphasize local structure, MACCS keys can detect pivotal substructures associated with toxicity, and Mordred descriptors offer an extensive feature space detailing global and electronic aspects of the molecule. The RDKit Python package (version 2024.03.5) was used to generate Morgan fingerprints with a radius of 2 and a length of 2048 bits, MACCS keys yielding a 166-bit vector, and Mordred descriptors totaling approximately 1600 features. Any Mordred descriptors with missing values were removed to ensure data quality and consistency. Next, a low-variance filter was employed to eliminate descriptors that contributed minimally to the target variable. Following this, the remaining features were scaled using scikit-learn’s StandardScaler, thereby standardizing features to a uniform range and reducing the impact of magnitude differences across variables. A combination of univariate and multivariate selection methods was then applied to pinpoint the most informative features for each specific endpoint. First, an Analysis of Variance (ANOVA) was performed to identify descriptors with statistically significant differences between classes (e.g., sensitizers vs. non-sensitizers). Subsequently, L1-penalized linear regression (also known as Lasso regression) was used to further reduce descriptor complexity by imposing sparsity, effectively zeroing out less relevant coefficients and producing a concise set of Mordred descriptors [51]. This two-step feature selection process ensured that only the most pertinent descriptors were retained for modeling, thereby mitigating issues arising from high dimensionality and helping minimize overfitting risks and computational overhead.

### 2.3 Dataset partitioning

**Fig. 1:**
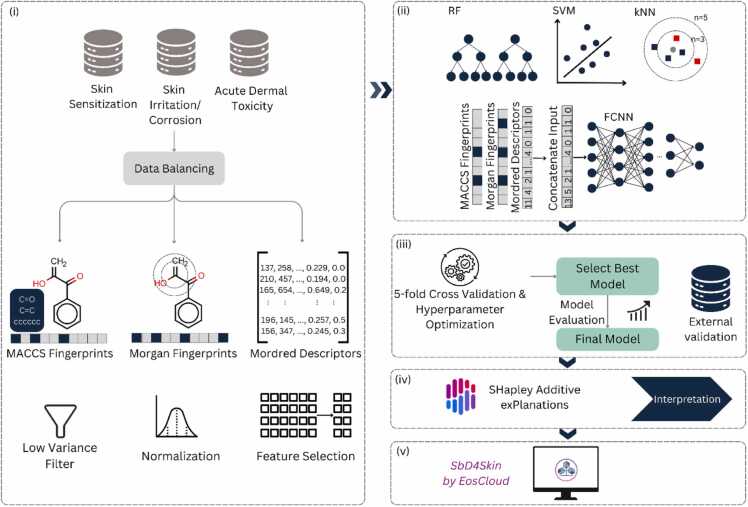
Overview of the study workflow. Our study pipeline consists of the following key steps: (i) data pre-processing, including data balancing, molecular featurization and feature pre-processing, (ii) model construction, (iii) model optimization, selection, and final model evaluation on test data and on external validation data, (iv) model interpretation, and (v) dissemination of the models as user-friendly tools via EosCloud. In data pre-processing, we used under-sampling methods to balance datasets, and split data to train, validation and test set. Then, three molecular representations were employed (Morgan fingerprints, MACCS keys, Mordred descriptors). These representations are used individually and in combination for model training. Feature pre-processing contains low variance filtering, normalization, and feature selection for Mordred descriptors. Then, four classifiers are employed, including 3 machine learning models and one deep learning model. Each model undergoes 5-fold cross validation to optimize its hyperparameters. The final model evaluation is performed on test dataset and on external dataset. Using SHAP the most important features for each skin toxicity endpoint are retrieved.

In the development of QSAR models, a widely accepted practice involves partitioning the dataset into training, validation, and test sets to ensure reliable model performance estimates. In this study, the entire dataset was first divided into training and test subsets in a 9:1 ratio. The test set was employed only at the final stage, after the model had been fully optimized, to evaluate its predictive capacity on previously unseen compounds. The remaining data were reserved for hyperparameter optimization via 5-fold stratified cross validation. Specifically, these data were split into five folds; in each iteration, four folds served as training data, and the remaining fold was used for validation. This procedure was repeated five times, making certain that each fold was used exactly once as the validation set. The model’s average performance across the five folds was then utilized to select the best hyperparameter configuration. Fig. 1 provides a schematic representation of this overall analysis workflow, and details concerning each step appear in subsequent paragraphs.

### 2.4 Model construction

Three ML algorithms and one fundamental DL algorithm were explored in this study, employing the feature spaces derived from MACCS keys, Morgan fingerprints, and Mordred descriptors. The methods included RF, SVM, kNN, and FCNN. RF is based on an ensemble of decision trees, where the final classification is determined by majority voting among individual trees. SVM locates an optimal hyperplane in the feature space, effectively separating different classes based on their characteristic properties. kNN predicts class membership by assessing the proximity of a new data point to existing, labeled points in the feature space. FCNNs are a form of DL architecture composed of multiple layers of interconnected neurons, allowing them to learn complex, non-linear patterns in the data. For the ML classifiers, systematic hyperparameter optimization was conducted using grid search aligned with the 5-fold cross validation approach, using accuracy as the principal objective function. The FCNN hyperparameters were optimized through the Hyperopt Python package [52]. To prevent overfitting FCNN models, we implemented several regularization techniques. Specifically, we applied dropout layers to randomly exclude neurons during training, batch normalization to stabilize convergence, and early stopping based on the validation loss to stop training before overfitting.

### 2.5 Model evaluation

The performance of all models was gauged using six key metrics: accuracy (ACC), Area Under the Receiver Operating Characteristic Curve (AUC-ROC), precision (Pre), specificity (Spe), sensitivity (Sen), and F1-score. These metrics provide complementary insights into different aspects of classification performance. The ACC, Pre, Spe, Sen, and F1 metrics were computed according to (1), (2), (3), (4), (5), where TP, FP, TN, and FN stand for true positives, false positives, true negatives, and false negatives, respectively. The AUC-ROC values were calculated based on the area under the curve between sensitivity and 1-specificity values. By comparing models against these multiple performance criteria, a robust evaluation of model strengths and weaknesses was achieved.

(1) $$\mathrm{Acc}=\frac{\mathrm{TP}+\mathrm{TN}}{\mathrm{TP}+\mathrm{TN}+\mathrm{FP}+\mathrm{FN}}$$

(2) $$\mathrm{Pre}=\frac{\mathrm{TP}}{\mathrm{TP}+\mathrm{FP}}$$

(3) $$\mathrm{Spe}=\frac{\mathrm{TN}}{\mathrm{TN}+\mathrm{FP}}$$

(4) $$\mathrm{Sen}=\frac{\mathrm{TP}}{\mathrm{TP}+\mathrm{FN}}$$

(5) $$F1=\frac{\mathrm{TP}}{\mathrm{TP}+0.5(\mathrm{FP}+\mathrm{FN})}$$

To verify the reliability of the models, we also performed Y-randomization tests. This method involves randomly swapping the endpoint values and developing new models. If the Y-randomization models perform poorly compared to the original model, then the original model contains a true correlation. We conducted Y-randomization tests for the final models of each endpoint, including skin sensitization, skin irritation/corrosion, and acute dermal toxicity, and the results are available in the corresponding QMRF documents.

### 2.6 Applicability domain

The concept of the applicability domain (AD) is fundamental for determining the reliability of QSAR predictions in a given chemical space [22]. Regulatory authorities often require explicit AD definitions for QSAR models, thus ensuring that predictions are only made for compounds structurally similar to those included in the training set. Several AD methodologies exist, including Euclidean distance-based and leverage-based methods, which are particularly suitable for descriptor-based features like Mordred descriptors [53]. Conversely, fingerprint-based approaches frequently adopt Tanimoto similarity thresholds to determine AD boundaries [54]. In this study, two approaches were used: a Euclidean distance-based method for Mordred descriptor features and a Tanimoto similarity-based method for MACCS keys and Morgan fingerprints [32, 53].

For the Euclidean distance-based AD, the centroid of the training compounds was calculated as the mean of each scaled descriptor. The distances between all training compounds and the centroid were calculated, and the largest distance was adopted as the threshold $d_{t}$. Then, the Euclidean distance $d_{\mathrm{Euc},i}$ between the test compound i and the centroid was calculated according to Eq. (6):

(6) $$d_{\mathrm{Euc},i}=\sqrt{\sum {\left(x_{j,i}-x_{j,\mathrm{centroid}}\right)}^{2}}$$

where $x_{j,i}\;$is the $j^{\mathrm{th}}$ scaled descriptor of compound i and $x_{j,\mathrm{centroid}}$ is the $j^{\mathrm{th}}$ scaled descriptor of the centroid. In case the $d_{\mathrm{Euc},i}$ of compound i is larger than the $d_{t}$, the compound is considered outside the AD.

The similarity-based AD was adopted for the fingerprint representation of compounds. This methodology computes the similarity matrix between the training compounds and the test compounds. A similarity threshold is calculated as defined in Eq. (7):

(7) $$S_{T}=\overset{®}{\gamma }+Ζ\sigma$$

where $\overset{®}{\gamma }$ represents the average Tanimoto similarity between training compounds, σ is the standard deviation of the computed Tanimoto similarities, and Z is a predefined value indicating the significance level. If the average Tanimoto similarity between the test compound and its k most similar compounds in the training set exceeds the threshold $S_{T}$, it is consided to be in the AD, otherwise the compound is considered outside the AD.

For models trained solely on Mordred descriptors, we utilized the Euclidean distance-based AD, calculating the distance between the query compound and the centroid of the training compounds. For fingerprint-based models, we employed the Tanimoto similarity-based AD approach, determining whether a compound falls within the applicability domain based on its similarity to the training compounds. For the models trained on concatenated features -combining Mordred descriptors and fingerprints- we considered both approaches independently to evaluate their individual contribution to the AD. Specifically, we applied the Euclidean distance-based approach to the descriptor subset and the similarity-based approach to the fingerprint subset, both MACCS and Morgan fingerprints. To determine whether a compound falls within the AD of the model, we adopted a consensus approach, considering a compound inside the AD if it meets at least two of the three defined thresholds. This strategy ensures a balanced assessment of prediction reliability and accounts for variations in molecular representations.

### 2.7 Dataset and model FAIRification and availability

As per the FAIR data principles [38], the enriched datasets, used for developing the models presented in this paper, have been uploaded in a ready-for-modelling format into the ChemPharos database (https://db.chempharos.eu/datasets/Datasets.zul) [42]. The compounds assigned to the training and test sets are explicitly annotated to ensure reproducibility. Compounds that were removed during the under-sampling process are also available in the ChemPharos database for each dataset. The datasets have been FAIRified for future reuse and for data and model transparency purposes, using the FAIR data interpretations by the GO FAIR Foundation [39] and the EU’s Joint Research Centre (JRC) Guidelines for FAIR Data [55]. In short, the data and metadata have been added into templates and assigned unique identifiers. The metadata describing the entire dataset has been catalogued as a machine-actionable nanopublication and published in nanodash [56]. The nanopublications for skin sensitization, skin irritation/corrosion, and acute dermal toxicity are available in [57, 58, 59]. The datasets are also available in machine actionable format, i.e., XML, for remote retrieval.

As model development and subsequent translation to software relies on data, but it's not data itself, the FAIR Data Principles cannot be fully applied. For example, while interoperability can be defined for the data used as input to train the model and the produced predictions, it cannot be defined for the model or the software itself. Therefore, the models have been released through a web application (https://eoscloud.entelos.eu/ssbd4chem/sbd4skin/) and have been partly FAIRified based on the guidelines for the FAIRification of computational workflows [60].

As per Wilkinson *et al*. (2025) [60] the models have been assigned a unique URI through the EosCloud Platform [61]. The models have been complemented with an API for remote access (https://eoscloud.entelos.eu/ssbd4chem/swagger-ui/index.html). Rich metadata have also been linked to it with the use of the QMRF template [40] and MODA through easy-MODA [41], which provides a standardized structure for documenting data-driven models and their predictions. It includes the model’s metadata like model identity, algorithm description, applicability domain, input descriptors, endpoint relevance, performance metrics, and uncertainty estimation. QMRF is in line with the OECD guidelines for the validation of QSAR models [40] and assists with model transparency, reproducibility, and regulatory acceptance. This approach makes the models, and the data used to produce them findable, accessible, interoperable, and reusable, avoiding black box practices and supporting integration into broader research and regulatory frameworks.

## 3 Results and discussion

In this study, we evaluated the performance of QSAR classification models for skin sensitization, skin irritation/corrosion and acute dermal toxicity. We used different featurization methods for our datasets, including MACCS keys, Morgan fingerprints, Mordred descriptors, and an integration of the above. We trained three machine learning classifiers, including RF, SVM, and kNN in each featurization method, to determine which combination yields the best predictive performance. A combination of univariate and multivariate methods was used for selecting the Mordred descriptors. For skin sensitization, 63 descriptors were selected as important for model training, while for skin irritation/corrosion and acute dermal toxicity prediction the feature selection process identified 62 and 69 descriptors, respectively. The concatenated vectors containing both fingerprints and selected descriptors were used to train an FCNN. The best-performing model for each case was identified based on the evaluation metrics during cross validation.

### 3.1 Hyperparameter optimization

For the ML classifiers, we conducted hyperparameter optimization using grid search aligned with the 5-fold cross validation approach, while for optimizing the hyperparameters of FCNN we utilized the Hyperopt Python package [52]. Table 1 lists the main hyperparameter settings investigated for RF, SVM, and kNN, while Table 2 presents the main hyperparameter ranges considered for the FCNN. The final, optimized hyperparameters for each model and featurization method combination appear in Table S1 of the Supplementary Material.

**Table 1:** Hyperparameter settings for random forest, support vector machines and k-nearest neighbors.

**Model**	**Hyperparameter description**	**Values**
Random Forest	Number of estimators	50, 100, 200
	Maximum depth	None, 5, 10, 20
	Minimum samples split	2, 5
Support Vector Machine	C	0.1, 1, 10, 100
	kernel	linear, poly, rbf, sigmoid
k-Nearest Neighbors	Number of neighbors	3, 5, 7, 9, 11
	Weight function	uniform, distance
	Metric for distance calculation	euclidean, manhattan

**Table 2:** Hyperparameter settings for fully connected neural network.

**Hyperparameter name**	**Hyperparameter description**	**Search space**
Learning rate	Learning rate for Adam optimizer	10^−5^ to 10^−2^
Batch size	Batch size of training data	32, 64
Dense Layer size 1	Number of nodes of the first dense layer	256, 512, 1024
Dense Layer size 2	Number of nodes of the second dense layer	64, 128, 256
Dropout 1	Dropout rate for the first dropout layer after the first dense layer	0.1, 0.2, 0.3, 0.4, 0.5
Dropout 2	Dropout rate for the second dropout layer after the second dense layer	0.1, 0.2, 0.3, 0.4, 0.5

### 3.2 Performance evaluation

We examined the effectiveness of individual ML models on different featurization methods, to identify which classifier yields better predictive accuracy. The performance metrics include accuracy, AUC-ROC, precision, specificity, sensitivity, and F1-score. Table S2, Table S3 and Table S4 summarize the performance of the QSAR classification models for skin sensitization, skin irritation/corrosion and acute dermal toxicity, respectively.

**Fig. 2:**
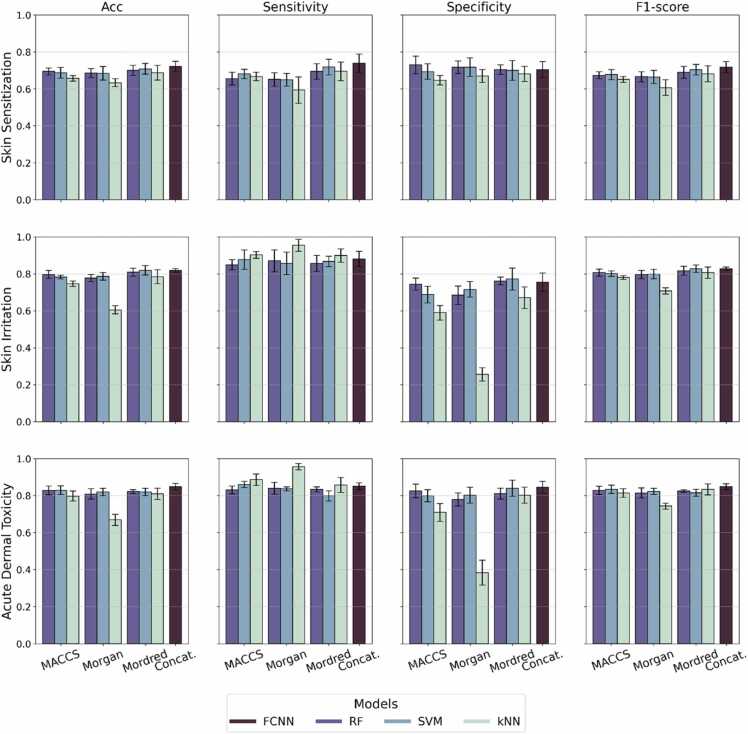
Evaluation metrics, including accuracy, sensitivity, specificity and F1-score for skin sensitization, skin irritation/corrosion, and acute dermal toxicity prediction using fingerprints, descriptors, and combined vectors. Difference colors represent different classifiers, including Random Forest, Support Vector Machine, k-Nearest Neighbors, and Fully Connected Neural Network.

The performance of QSAR classification modelling for skin sensitization shows slight differences when using MACCS or Morgan fingerprints. The best model using fingerprint-based features on 5-fold cross validation is achieved with RF and MACCS keys, resulting in an accuracy of 0.69 ± 0.02 and an AUC of 0.75 ± 0.02. Across both fingerprint types, kNN underperformed compared to RF and SVM. Models using Mordred descriptors generally outperformed fingerprint-based models. The best performing model was the SVM classifier, which achieved an accuracy of 0.71 ± 0.03 and an AUC of 0.77 ± 0.04. The combination of MACCS keys fingerprints, Morgan fingerprints and Mordred descriptors passed through an FCNN resulted the best overall performance with an accuracy on cross validation of 0.72 ± 0.03 and an AUC of 0.79 ± 0.02. A visualization of the performance metrics, including AUC, sensitivity, specificity, and F1-score, using different featurization methods and classifiers is presented in Fig. 2.

**Fig. 3:**
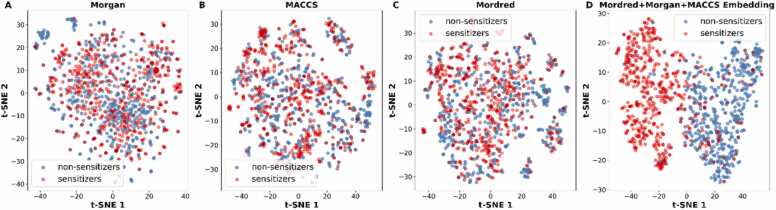
Molecular features distribution of the skin sensitization dataset using (A) Morgan fingerprints, (B) MACCS keys, (C) Mordred descriptors and (D) the molecular embeddings of the concatenated vectors.

We further utilized the t-SNE algorithm to visualize the chemical distribution with different molecular representations. As shown in Fig. 3, there is a small degree of discrimination between sensitizers and non-sensitizers using Morgan fingerprints, MACCS keys, or Mordred descriptors. However, the embeddings of the FCNN model using the concatenated vectors show a clear distinction of the classes, suggesting the potential of concatenated vectors to distinguish between sensitizers and non-sensitizers.

**Fig. 4:**
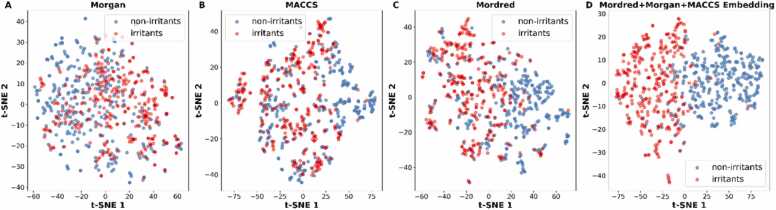
Molecular features distribution of the skin irritation dataset using (A) Morgan fingerprints, (B) MACCS keys, (C) Mordred descriptors and (D) the molecular embeddings of the concatenated vectors.

For the skin irritation/corrosion, using fingerprints and molecular descriptors separately (Table S3) achieves the best accuracy values of 0.80 ± 0.02 and 0.82 ± 0.03, respectively. The models selected are RF using MACCS fingerprints and SVM using Mordred descriptors. When combining the fingerprints and descriptors, FCNN results in similar performance, with an accuracy of 0.82 ± 0.01 and AUC 0.88 ± 0.01. Across the best models, the precision values range from 0.77 to 0.79, the specificity range from 0.74 to 0.77, the sensitivity range from 0.85 to 0.88, and F1 score range from 0.81 to 0.83. In skin irritation dataset, combining different descriptors using FCNN did not significantly improve the overall performance but provided comparable results with a balanced metric distribution. The t-SNE visualization of the skin irritation dataset for all molecular representations are shown in Fig. 4. The molecular embeddings show a clear separation of the compounds as irritants and non-irritants.

**Fig. 5:**
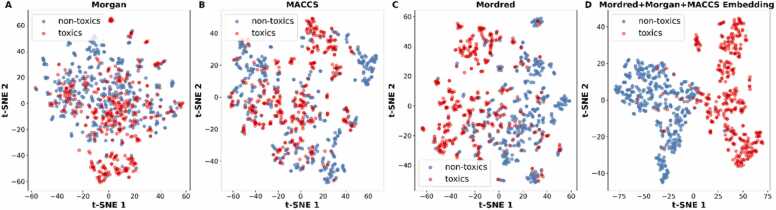
Molecular features distribution of the acute dermal toxicity dataset using (A) Morgan fingerprints, (B) MACCS keys, (C) Mordred descriptors and (D) the molecular embeddings of the concatenated vectors.

Similarly, for acute dermal toxicity, we selected SVM with MACCS fingerprints and RF with Mordred descriptors. The fingerprint-based model achieved an accuracy of 0.83 ± 0.02 and an AUC of 0.90 ± 0.02. Using Morgan fingerprints, kNN shows the highest sensitivity of 0.96 but suffers from poor specificity. The descriptor-based model achieved an accuracy of 0.82 ± 0.01 and an AUC 0.90 ± 0.03. The FCNN model, integrating all three feature sets, achieved the best performance with an accuracy of 0.85 ± 0.02 and an AUC of 0.92 ± 0.02. The FCNN embeddings using the concatenated vectors of Morgan fingerprints, MACCS keys, and Mordred descriptors, show a clear distinction of compounds based on their toxicity (Fig. 5). A less strong separation is achieved using Mordred descriptors, while the fingerprint-type representation did not show a strong ability to separate the classes.

The combination of fingerprints and molecular descriptors consistently produce similar predictions of the best individual model (e.g., SVM with Mordred descriptors in case of skin irritation/corrosion) or even better predictions. Based on these findings, we selected FCNN as the final model for all endpoints, as it demonstrated robust performance across different tasks. These results highlight the value of combining featurization methods for better capturing molecular patterns, improving overall predictive power.

We further validated the performance of FCNN on a test set, which consisted of 10 % of the original dataset. This test set was held out during training to assess model generalizability on unseen data from the same distribution. Table 3 summarizes the performance on the test set across three endpoints: skin sensitization, skin irritation/corrosion, and acute dermal toxicity. All three models demonstrate strong predictive capabilities on the test set with an AUC of 0.88 for skin sensitization, 0.91 for skin irritation and 0.82 for acute dermal toxicity. Furthermore, the models demonstrate balance between specificity and sensitivity, with values on specificity ranging from 0.86 to 0.87 and values on sensitivity ranging from 0.72 to 0.84. Therefore, all three selected models for each toxicity endpoint performed robust results on unseen data.

**Table 3:** Performance of FCNN on the test dataset.

Endpoints	Acc	AUC	Pre	Spe	Sen	F1
Skin Sensitization	0.85	0.88	0.85	0.87	0.83	0.84
Skin Irritation/Corrosion	0.86	0.91	0.87	0.86	0.84	0.86
Acute Dermal Toxicity	0.79	0.82	0.85	0.87	0.72	0.78

### 3.3 Feature importance analysis

To comprehensively evaluate the QSAR models and gain a better understanding of their functioning, we conducted an in-depth feature importance analysis. We performed a SHAP (Shapley Additive exPlanations) analysis to interpret the predictions of the FCNN models for skin sensitization, skin irritation/corrosion, and acute dermal toxicity [62]. SHAP is a game theoretic approach to explain the output of a ML model, by calculating the marginal contributions of features to the output of the model. The input model is defined as the linear addition of the input variables [63]. The model output f(x) can be defined as:

(8) $$f\left(x\right)=g\left(x^{\prime }\right)=\varphi_{0}+\sum_{i=1}^{M}\;\varphi_{i}x_{i}^{\prime }$$

where $g(x^{\prime })$ is the interpretation model of the simplified input x′, $\varphi_{0}$ is a constant value when all features are missing and $\varphi_{i}$ is the feature attibution of a feature i. SHAP methods can be divided into Tree SHAP, Kernel SHAP and Deep SHAP. In this study, Deep SHAP is used on training data to explain the FCNN model.

**Fig. 6:**
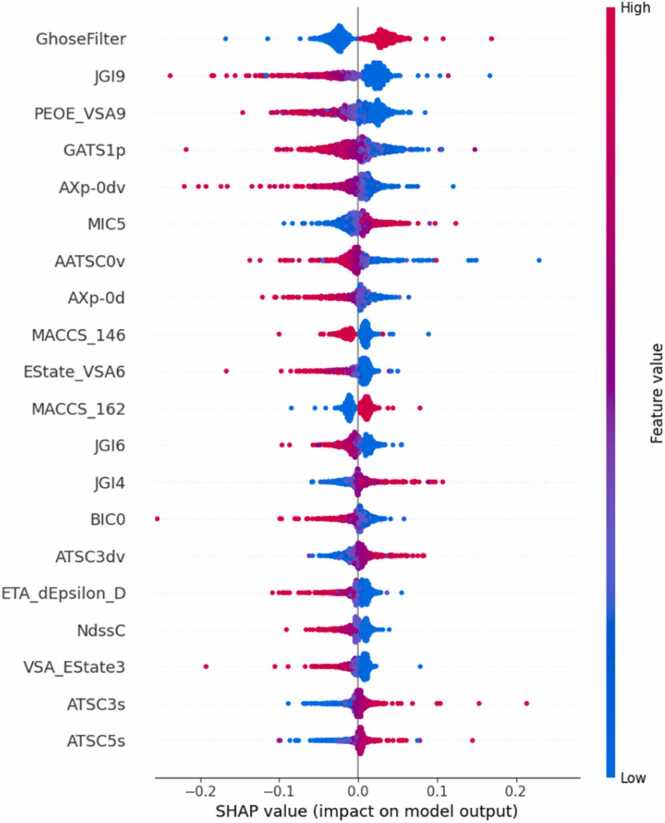
SHAP analysis for concatenated vectors of MACCS keys, Morgan fingerprints and Mordred descriptors for skin sensitization.

**Fig. 7:**
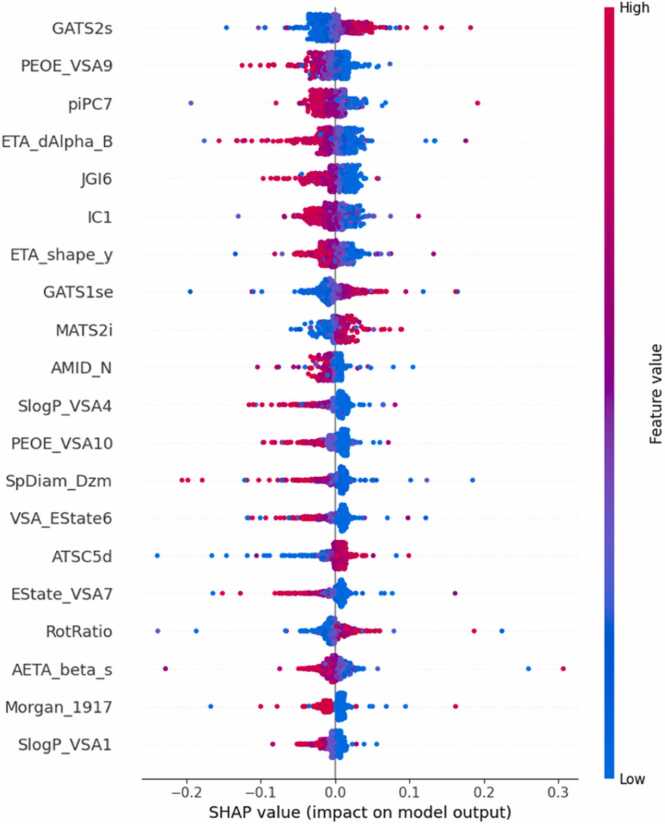
SHAP analysis for concatenated vectors of MACCS keys, Morgan fingerprints and Mordred descriptors for skin irritation/corrosion.

**Fig. 8:**
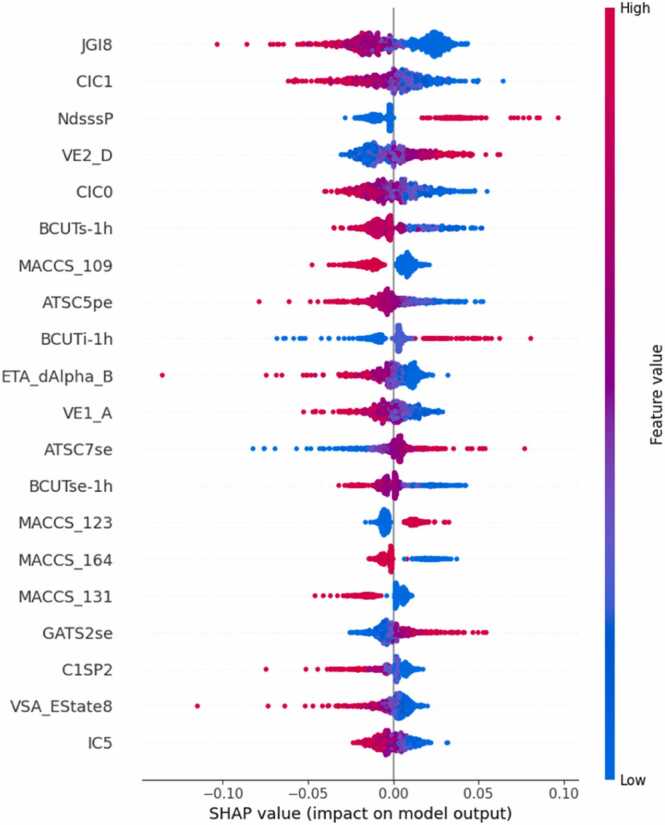
SHAP analysis for concatenated vectors of MACCS keys, Morgan fingerprints and Mordred descriptors for acute dermal toxicity.

A feature analysis for skin sensitization is presented in Fig. 6, for skin irritation in Fig. 7, and for acute dermal toxicity in Fig. 8. The most influential features for predicting skin sensitization from MACCS keys fingerprints were MACCS_162 which refers to the presence of sulfur-containing groups (e.g. thiols, sulfides, and sulfonates) and MACCS_146, which refers to nitrogen-containing groups [64]. While sulfur is widely used for treating dermatological conditions [65], its presence is also linked to mild sensitization. SHAP analysis reveals that high values of sulfur containing compounds correspond to greater likelihood of skin sensitization. Despite the fact that the presence of nitrogen containing groups is related to skin sensitization due to their ability to form reactive intermediates that bind covalently to skin proteins [66], SHAP analysis did not identify this feature as correlated with skin sensitization. This might be due to the fact that the presence of nitrogen-containing groups is not always a clear indicator of sensitization and certain types of nitrogen-containing groups might be less sensitizing. Utilizing Mordred descriptors, the most influential features include Ghose Filter, JGI9, PEOE_VSA9, and GATS1p. GATS1p is a Geary Auto-Correlation feature that reflects the polarizability between atoms that are one bond apart in the molecule. PEOE_VSA9 captures the partial equalization of orbital electronegativities across molecular surfaces and descriptors like JGI9 and JGI6 relate to topological charge indices, which are indicative of the overall electronic distribution within the molecule. Lower values of these descriptors are generally associated with non-sensitizers, aligning with existing literature that links molecular polarity and charge distribution to sensitization potential [67]. The Ghose Filter was found to influence skin sensitization. Since Ghose Filter looks at factors like molecular weight, lipophilicity, rotatable bonds and hydrogen bond donors and acceptors, it may contribute to skin sensitization since lower molecular weight might penetrate the skin and cause sensitization, lipophilic compounds might indicate potential skin absorption, and more hydrogen bonding might be an indicator of more reactive compounds.

The 20 most influential features for predicting skin irritation are displayed in Fig. 7. GATS2s is another Geary Auto-Correlation feature, which reflects a spatial correlation between atom types and their properties when atom pairs are two bonds apart. This descriptor focuses on electrostatic properties, and it reflects the distribution of charges across the molecule. A higher value of GATS2s indicates grater spatial variations of the electrostatic properties across the molecule, which might enhance its reactivity and cause skin irritation [68]. The SHAP analysis identified this descriptor as highly correlated with skin irritation prediction. Furthermore, higher values of GATS1se, RotRatio and MATS2i were found to correspond to a greater likelihood of skin irritants. Similar to GATS2s, GATS1se is a Geary Auto-Correlation feature that reflects the distribution of electrostatic properties between atoms that are one bond apart in the molecule. Molecules with distinct charge distribution may form electrostatic interactions with skin proteins and cause skin irritation. Higher RotRatio, meaning molecules with higher rotatable bond ratio implies a more flexible molecule, which can penetrate skin and interact with skin proteins, increasing the likelihood of irritation. Lastly, MATS2i, the Moran coefficient of lag 2 weights by ionization potential, focuses on the electron distribution of the molecule and can potentially indicate acidic properties or electronic centers that are reactive, causing skin irritation [68].

For acute dermal toxicity, higher values of NdsssP, VE2_D, BCUTi-1h, ATSC7se, and GATS2se features were found to move towards higher likelihood of dermal toxicants (Fig. 8). MACCS_123, MACCS_131 and MACCS_164 were identified as important fingerprint-based features for predicting dermal toxicity. NdsssP refers to the number of atom type dsssP, VE2_D to the average coefficient of the last eigenvector from topological distance matrix, BCUTi-1h to the first highest eigenvalue of Burden matrix weighted by ionization potential, ATSC7se to the moreau-broto autocorrelation of lag 7 weighted by Sanderson electronegativity and GATS2se, similar to GATS1se is a Geary coefficient of lag 2. NdsssP and BCUTi-1h capture the presence of reactive groups and electronic properties respectively, which can lead to skin toxicity. Similarly, the higher value of ATSC7se and GATS2se might indicate a specific pattern of electronegativity distribution that enhances skin penetration or reactivity.

### 3.4 Model validation with known toxicants

To further assess the robustness of our models, we conducted additional external validation using known toxicants collected from the literature. These datasets consist of compounds absent from the training set, allowing us to evaluate the models’ predictive performance on truly unseen data, and assess models’ performance on real case scenarios. While evaluating model performance on non-toxicants is also important, correctly identifying toxicants is crucial in the early stages of compound filtering. Accurate prediction of toxic compounds facilitates their early removal, streamlining subsequent steps and minimizing the risk of advancing harmful candidates.

For skin sensitization, a list of 45 potential skin sensitizers in cosmetic ingredients was compiled by the Norwegian Scientific Committee for Food Safety [69]. Of these, 11 were absent from the skin sensitization dataset. A previous study by Borba *et al*. used this dataset for model validation, resulting in predicting 8 out of 11 toxicants correctly [33]. Our model identified 9 out of 11, resulting in sensitivity of 0.82. Additionally, we tested our model on an external dataset compiled by the Cosmetics Europe consortium [70, 71]. Since this dataset contained compounds classified as non-sensitizers, weak, moderate and strong sensitizers, we grouped weak, moderate and strong sensitizers into one class, the sensitizers class. A list of 95 sensitizers was compiled, and 37 were absent from the skin sensitization dataset. Our model identified 32 out of 37 sensitizers, resulting in a sensitivity of 0.86 (Table 4).

**Table 4:** External datasets of known toxicants and their prediction through SbD4Skin.

Endpoints	Source	Toxicants	Sensitivity
Skin Sensitization	Norwegian Scientific Committee for Food Safety [69]	11	0.82
	Cosmetics Europe Database [70], [71]	37	0.86
Skin Irritation/Corrosion	ChemSkin Database [72]	263	0.89
Acute Dermal Toxicity	Literature [33]	2	1.00
	NITE [73]	132	0.86

For skin irritation, we collected known skin irritants used in the study of Kang *et al*. [31] which were collected from the ChemSkin Database [72]. Additionally, Borba *et al*. [33] proposed two known irritants, one of which was already in ChemSkin Database, so we only added the MS-222 compound, a fish anesthetic commonly used in aquaculture. After filtering out compounds that were already present in our training set, the external validation dataset consisted of 263 known skin irritants. Our model correctly identified 233 out of 263 skin irritants, resulting in a sensitivity of 0.89.

The model predicting acute dermal toxicity was also validated using an external validation set. The compounds dichloromethane and methanol have been reported as systemic toxicants after dermal exposure and were used for validating Borba *et al*. [33] model. Our model correctly predicted both compounds as toxic after dermal exposure. We also collected dermal toxic compounds from NITE [73]. After removing common compounds between training and external validation dataset, the final dataset consisted of 132 dermal toxicants. Our model correctly identified 113 out of 132 toxicants, achieving a sensitivity of 0.86.

The predictions for these known toxicants (Table 4) aligned well with their expected toxicity labels, demonstrating that our models generalize well beyond the training data, making accurate predictions for unseen compounds.

### 3.5 Applicability domain analysis

To ensure the reliability of our model predictions, we conducted a domain of applicability (AD) analysis. Since our model utilized MACCS keys, Morgan fingerprints and Mordred descriptors, we used two similarity-based approaches for fingerprint-type representations and one Euclidean distance-based approach for descriptor-type representation. The compounds are considered inside the AD if they meet at least two of the three defined thresholds. As presented above, we validated our model both on test set (10 % split derived from the original training dataset) and on completely external validation using literature data. We calculated the AD for test set and external validation set, and we evaluated our models on compounds inside the AD and outside the AD. For skin sensitization, compounds in test dataset and known sensitizers from literature are considered inside the AD. For skin irritation, two compounds from known irritants from literature are considered outside AD. Despite being outside the AD, these two compounds were correctly classified as irritants. For acute dermal toxicity, one compound from the test dataset was outside the AD and it was correctly predicted as toxic after dermal exposure. While our current reliability estimation is based on chemical coverage and AD, we acknowledge that other approaches, such as probability-based or distance to the model, have also been shown to provide improved model performance [34]. Future versions of the models may incorporate such methods to complement our current AD-based reliability approach.

**Fig. 9:**
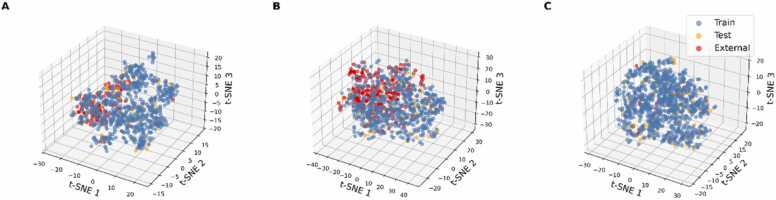
Three-dimensional t-distributed stochastic neighbor embedding (t-SNE) plot of training set, test set, and external validation set (known toxicants) of (A) skin sensitization dataset, (B) skin irritation/corrosion dataset, and (C) acute dermal toxicity dataset. The t-SNE analysis was performed by employing Mordred descriptors.

To further validate the consistency of the datasets, we employed Mordred descriptors to analyze the chemical space of the training set, test set and external validation set from the literature. Using t-SNE, we visualized these datasets in a 3D space as shown in Fig. 9. The t-SNE results demonstrate that all three datasets for each toxicity endpoint (skin sensitization, skin irritation, and acute dermal toxicity) share a similar chemical space. The compounds are well-described by the selected variables and cover the space uniformly to ensure reliable predictions.

### 3.6 Model implementation

The developed and validated models have been disseminated and made accessible via the SbD4Skin (Safe by Design for Skin) web application (https://eoscloud.entelos.eu/ssbd4chem/sbd4skin/), hosted in the EosCloud Platform [61]. The EosCloud Platform is an advanced suite of predictive modeling tools built using the ZK framework, JavaScript, and Java, offering ready-to-use web services for cheminformatics and nanoinformatics. The graphical user interface (GUI) allows users to input compounds of interest in three ways, either by drawing a molecule using an integrated sketcher, or uploading a molecule either in SMILES format or as Spatial Data File (SDF). Additionally, users can explore a 3D visualization of their molecular structures prior the energy minimization. The platform also supports high-throughput virtual screening, allowing users to investigate large datasets with a single query. The user can submit the molecules of interest and receive the predictions within seconds.

**Fig. 10:**
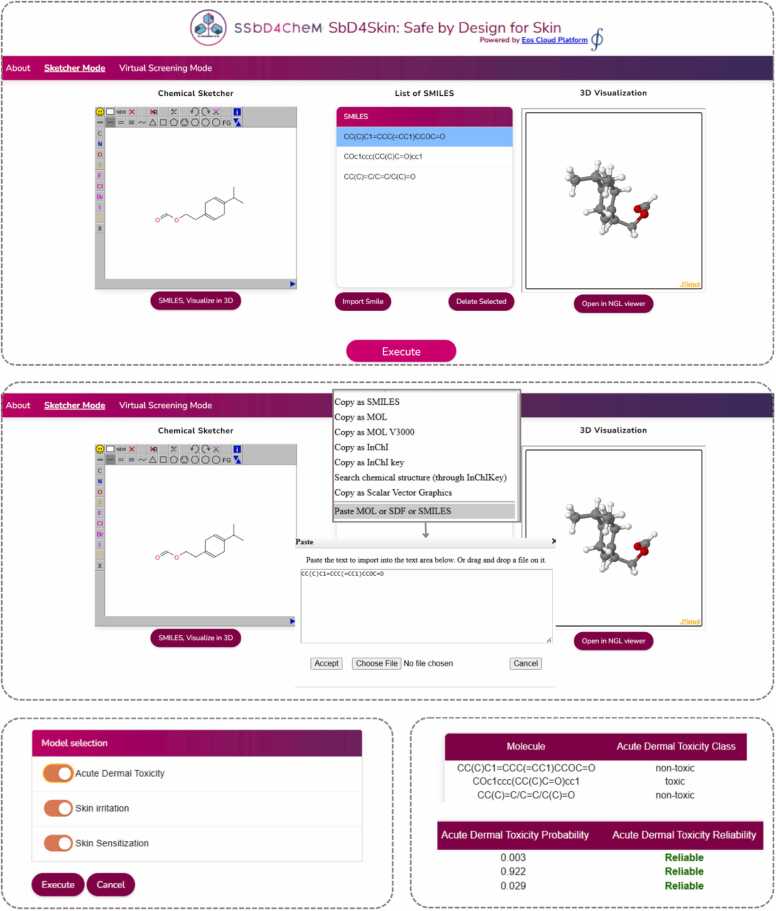
SbD4Skin GUI and output overview. The users can input query compounds by drawing them in the chemical sketcher, uploading them in SMILES format, or uploading them as SDF files. A 3D visualization option is also available. Then, the users select the endpoints from the list of the available models. For each query compound, predictions for all selected endpoints are presented, along with the reliability of the predictions.

The results include the predicted class for each toxicity endpoint (e.g., skin sensitizer or non-sensitizer), the probability of the prediction, and the reliability of the prediction with a color-coded indication, whereby green text indicates a reliable prediction while red text indicates an unreliable prediction. All results can be downloaded for further analysis, enhancing usability for diverse research applications. An overview of the SbD4Skin GUI is presented in Fig. 10.

Compared to other available tools, SbD4Skin provides a more scalable and user-friendly solution for toxicity prediction. For instance, STopTox allows submission of only one compound at a time, returning the predicted class, applicability domain and predicted fragment contribution per endpoint, which limits its use in batch screening workflows [33]. Similarly, SaferSkin is also restricted to assessing one compound at a time, it is limited to skin sensitization prediction and requires a commercial license for full access to its features [17, 74]. On the other hand, HuSSPred enables the submission of multiple compounds but is also restricted to skin sensitization prediction [27]. AttentiveSkin provides open-source code and integrates the classifiers into the admetSAR2 web tool, but is also limited to skin irritation/corrosion potentials [30]. Lastly, DermalPred focus solely on acute dermal toxicity and requires software installation [32]. In contrast, SbD4Skin combines multi-endpoint toxicity predictions, 3D visualization and batch processing in a single, accessible web interface, making it a practical solution for computational toxicology workflows. Predicting multiple endpoints is crucial because compounds can exhibit distinct toxicological profiles. For example, nicotine is acutely dermally toxic but not a skin irritant [75]. This finding aligns with predictions from SbD4Skin, which identified nicotine as toxic but not irritant. By addressing multiple endpoints simultaneously, SbD4Skin ensures a comprehensive safety assessment, accommodating cases where skin toxicity endpoints do not necessarily coincide.

The implementation also follows the FAIR principles for data, metadata, and the model itself. It offers programmatic access via APIs and allows data interoperability through machine-actionable formats, e.g., JSON, XML. The SbD4Skin platform provides downloadable results in both structured, ready-for-modelling formats and standardized reporting templates, e.g., QMRF, enhancing data traceability and reproducibility for research, industry, and regulatory users.

## 4 Conclusions

This research demonstrates the development of a robust computational framework for predicting skin sensitization, skin irritation/corrosion, and acute dermal toxicity using multi-view representations. We evaluated different molecular representation methods -MACCS keys, Morgan fingerprints and Mordred descriptors- with ML models, including RF, SVM and kNN. We then explored the integration of these representations into a unified input space for training an FCNN. The multi-view representation FCNN model achieved similar predictive accuracy to single-representation model on skin irritation and outperformed on skin sensitization and acute dermal toxicity models. External validation further confirmed the models’ robustness, with accuracy scores of 0.85, 0.86, and 0.79 for skin sensitization, irritation, and acute dermal toxicity, respectively. Notably, the models were also able to correctly identify known toxicants that were not included in the training set, with sensitivity values ranging from 0.82 to 0.89, demonstrating strong generalizability and real-word applicability. To ensure the reliability of predictions, we performed an AD analysis, demonstrating that most correctly classified compounds fell within the AD.

To enhance model interpretability, SHAP-based feature importance analysis was conducted for all three endpoints. For skin sensitization, sulfur-containing groups and descriptors related to polarity and electronic distribution emerged as influential. For skin irritation/corrosion, important features were related to electrostatic and topological properties. For acute dermal toxicity, important descriptors were linked to electronegativity distribution and reactivity. These findings provide valuable chemical insights, supporting the mechanistic relevance of selected features in skin toxicity prediction. Such knowledge can guide the early-stage design and screening of ingredients in the development of safer skin products by highlighting structural features associated with adverse effects.

Our models are implemented in SbD4Skin, a web-based tool designed for rapid and accessible toxicity predictions. SbD4Skin can be a practical and efficient tool to identify skin toxicity on compounds in early-stage screening workflows and to focus on promising candidates for various applications (e.g., in cosmetics products). This study highlights the effectiveness of multi-view molecular representations in advancing *in silico* toxicology, supporting regulatory frameworks, and promoting the adoption of NAMs to reduce reliance on animal testing. In line with the FAIR data principles, all data and models have been structured to ensure accessibility and reusability, with programmatic access and standardized documentation enabling integration into regulatory and scientific workflows.

## CRediT authorship contribution statement

**Nikoletta-Maria Koutroumpa:** Conceptualization, Methodology, Software, Validation, Formal analysis, Writing – original draft, Visualization, Writing – review & editing. **Dimitra-Danai Varsou:** Conceptualization, Methodology, Writing – review & editing, Supervision. **Panagiotis D. Kolokathis:** Methodology, Writing – review & editing, Funding acquisition. **Maria Antoniou:** Data curation, Writing – review & editing. **Konstantinos D. Papavasileiou:** Writing – review & editing. **Eleni Papadopoulou:** Software. **Anastasios G. Papadiamantis:** Writing – review & editing. **Andreas Tsoumanis:** Software. **Georgia Melagraki:** Writing – review & editing. **Milica Velimirovic:** Writing – review & editing, Project administration. **Antreas Afantitis:** Conceptualization, Writing – review & editing, Supervision, Funding acquisition.

## Declaration of Competing Interest

NovaMechanics is a cheminformatics and materials informatics company.

## Footnotes

Appendix A Supplementary data associated with this article can be found in the online version at doi:10.1016/j.csbj.2025.08.001.
