# Supplementary material for: SbD4Skin by EosCloud: Integrating multi-view molecular representation for predicting skin sensitization, irritation, and acute dermal toxicity
Source: Comput Struct Biotechnol J. 2025 Aug 6;29:222–35. doi: 10.1016/j.csbj.2025.08.001 (PMC12358665; doi:10.1016/j.csbj.2025.08.001)
Supplement: Supplementary file 1 — Supplementary material [file mmc1.docx]

**Supplementary Material**

SbD4Skin by EosCloud: Integrating Multi-View Molecular Representation for Predicting Skin Sensitization, Irritation, and Acute Dermal Toxicity

Nikoletta-Maria Koutroumpa ^1,2^, Dimitra-Danai Varsou ^3^, Panagiotis D. Kolokathis ^3^, Maria Antoniou ^1^, Konstantinos D. Papavasileiou ^3^, Eleni Papadopoulou ^1^, Anastasios G. Papadiamantis ^1^, Andreas Tsoumanis ^3^, Georgia Melagraki ^4^, Milica Velimirovic ^5^, Antreas Afantitis ^1,*^

^1^ Entelos Institute, Nicosia 2102, Cyprus

^2^ School of Chemical Engineering, National Technical University of Athens, 157 80 Athens, Greece

^3^ NovaMechanics MIKE, Piraeus 18545, Greece

^4^ Division of Physical Sciences & Applications, Hellenic Military Academy, 16672 Vari, Greece

^5^ Flemish Institute for Technological Research (VITO), Boeretang 200, 2400 Mol, Belgium

* Corresponding Author: afantitis@novamechanics.com

CONTENTS

[**Section 1:** t-SNE visualization for skin irritation/corrosion and acute dermal toxicity dataset following under-sampling procedure 3](#_Toc204783267)

[**Section 2:** Selected hyperparameters 4](#_Toc204783268)

[**Section 3:** Results on 5-fold cross validation 5](#_Toc204783269)

[**Section 4:** QMRF for skin sensitization model 6](#_Toc204783270)

[**Section 5:** QMRF for skin irritation/corrosion model 13](#_Toc204783271)

[**Section 6:** QMRF for acute dermal toxicity model 20](#_Toc204783272)

[**Section 7:** MODA for skin sensitization model 27](#_Toc204783273)

[**Section 8:** MODA for skin irritation/corrosion model 42](#_Toc204783274)

[**Section 9:** MODA for acute dermal toxicity model 56](#_Toc204783275)

# **Section 1:** t-SNE visualization for skin irritation/corrosion and acute dermal toxicity dataset following under-sampling procedure


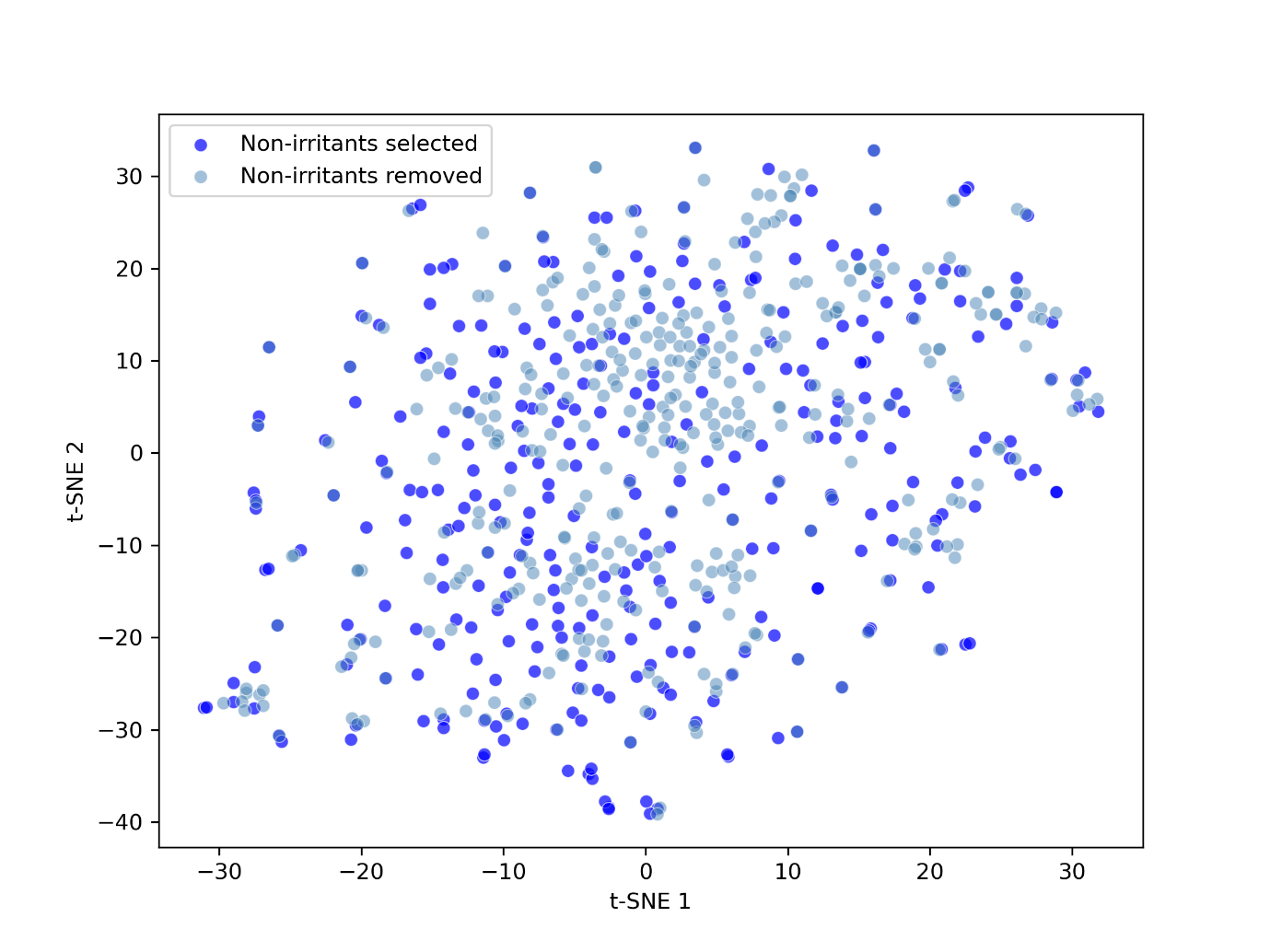


**Figure S1.** t-SNE visualization of retained and removed non-irritants based on Morgan fingerprint representations following under-sampling skin irritation/corrosion dataset.


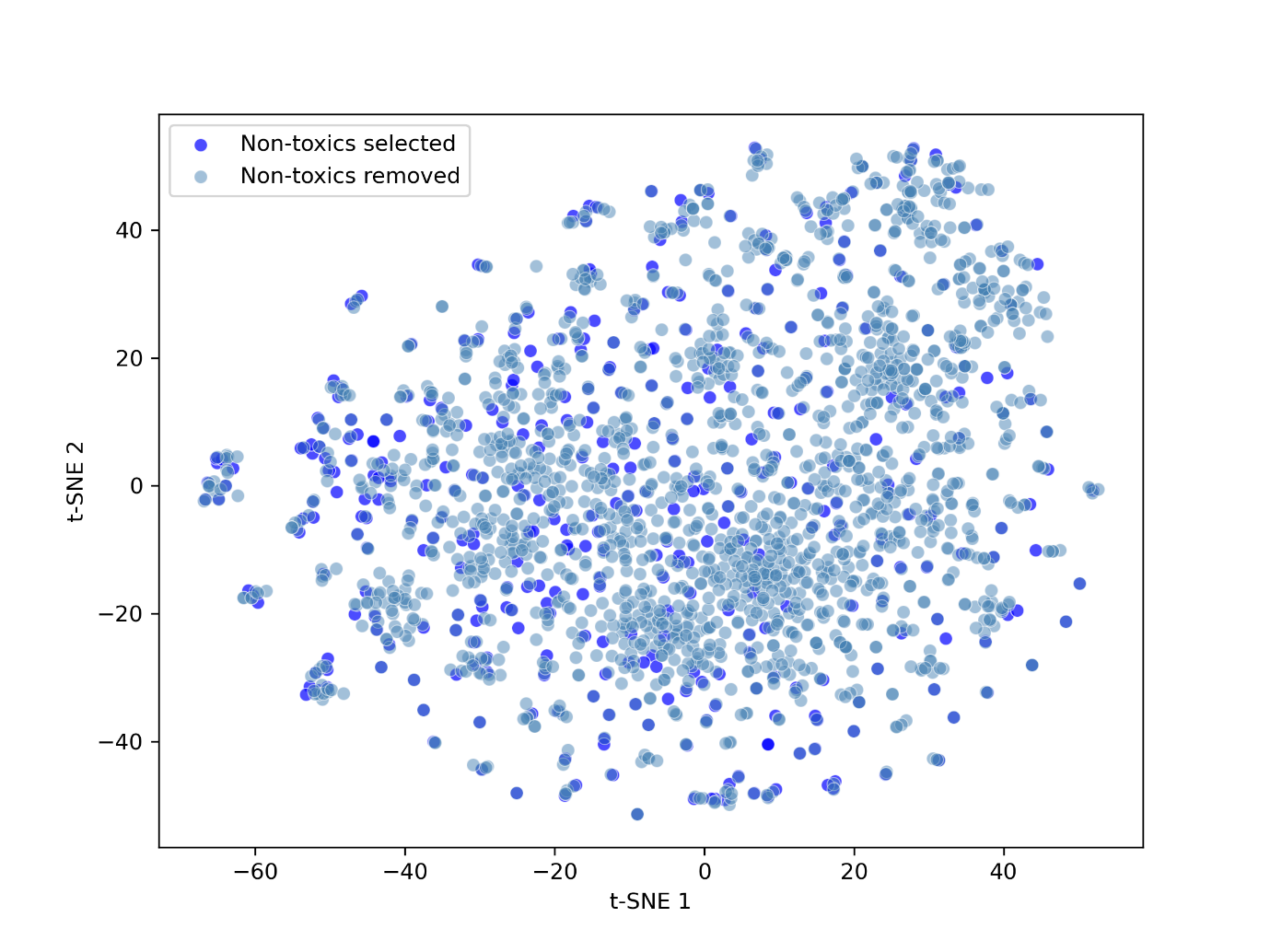


**Figure S2.** t-SNE visualization of retained and removed non-toxic compounds based on Morgan fingerprint representations following under-sampling acute dermal toxicity dataset.

# **Section 2:** Selected hyperparameters

**Table S1.** Selected hyperparameters for developed models.

| Model | Featurization method | Hyperparameter description | Skin sensitization | Skin irritation | Acute Dermal Toxicity |
| --- | --- | --- | --- | --- | --- |
| RF | MACCS keys | Number of estimators | 100 | 100 | 200 |
|  |  | Maximum depth | None | 10 | 20 |
|  |  | Minimum samples split | 5 | 5 | 5 |
|  | Morgan fingerprints | Number of estimators | 200 | 50 | 200 |
|  |  | Maximum depth | None | 10 | None |
|  |  | Minimum samples split | 2 | 5 | 5 |
|  | Mordred descriptors | Number of estimators | 200 | 200 | 50 |
|  |  | Maximum depth | 10 | None | None |
|  |  | Minimum samples split | 2 | 2 | 2 |
| SVM | MACCS keys | C | 1 | 1 | 1 |
|  |  | kernel | rbf | rbf | rbf |
|  | Morgan fingerprints | C | 10 | 1 | 10 |
|  |  | kernel | rbf | rbf | rbf |
|  | Mordred descriptors | C | 10 | 0.1 | 10 |
|  |  | kernel | linear | linear | poly |
| kNN | MACCS keys | Number of neighbors | 11 | 11 | 5 |
|  |  | Weight function | distance | uniform | distance |
|  |  | Metric for distance calculation | manhattan | euclidean | manhattan |
|  | Morgan fingerprints | Number of neighbors | 9 | 5 | 5 |
|  |  | Weight function | distance | manhattan | uniform |
|  |  | Metric for distance calculation | manhattan | euclidean | euclidean |
|  | Mordred descriptors | Number of neighbors | 9 | 11 | 7 |
|  |  | Weight function | distance | distance | distance |
|  |  | Metric for distance calculation | euclidean | euclidean | manhattan |
| FCNN | Concatenated vector | Learning rate for Adam optimizer | 10^-3^ | 10^-4^ | 10^-3^ |
|  |  | Batch size of training data | 32 | 32 | 32 |
|  |  | Number of nodes of the first dense layer | 1024 | 512 | 256 |
|  |  | Number of nodes of the second dense layer | 256 | 256 | 128 |
|  |  | Dropout rate for the first dropout layer after the first dense layer | 0.3 | 0.3 | 0.2 |
|  |  | Dropout rate for the second dropout layer after the second dense layer | 0.1 | 0.1 | 0.1 |

# **Section 3:** Results on 5-fold cross validation

**Table S2.** 5-fold cross validation performance comparison of skin sensitization prediction using different featurization methods.

| Feature | Model | Acc | AUC | Pre | Spe | Sen | F1 |
| --- | --- | --- | --- | --- | --- | --- | --- |
| MACCS | RF | 0.69±0.02 | 0.75±0.02 | 0.69±0.04 | 0.73±0.05 | 0.66±0.03 | 0.67±0.02 |
|  | SVM | 0.69±0.03 | 0.74±0.03 | 0.67±0.04 | 0.69±0.04 | 0.68±0.03 | 0.68±0.03 |
|  | kNN | 0.66±0.02 | 0.72±0.02 | 0.64±0.02 | 0.65±0.03 | 0.67±0.02 | 0.65±0.02 |
| Morgan | RF | 0.69±0.02 | 0.75±0.03 | 0.68±0.03 | 0.72±0.03 | 0.65±0.04 | 0.67±0.03 |
|  | SVM | 0.68±0.04 | 0.74±0.02 | 0.68±0.04 | 0.72±0.05 | 0.65±0.04 | 0.67±0.04 |
|  | kNN | 0.63±0.02 | 0.68±0.04 | 0.63±0.02 | 0.67±0.04 | 0.59±0.07 | 0.61±0.04 |
| Mordred | RF | 0.70±0.03 | 0.78±0.04 | 0.69±0.03 | 0.70±0.03 | 0.70±0.04 | 0.69±0.03 |
|  | SVM | 0.71±0.03 | 0.77±0.04 | 0.69±0.04 | 0.70±0.05 | 0.72±0.04 | 0.70±0.03 |
|  | kNN | 0.69±0.04 | 0.76±0.04 | 0.67±0.04 | 0.68±0.04 | 0.70±0.05 | 0.68±0.04 |
| Concat. Vectors | **FCNN** | **0.72±0.03** | **0.79±0.02** | **0.70±0.03** | **0.70±0.04** | **0.74±0.05** | **0.72±0.03** |

**Table S3.** 5-fold cross validation performance comparison of skin irritation/corrosion prediction using different featurization methods.

| Feature | Model | Acc | AUC | Pre | Spe | Sen | F1 |
| --- | --- | --- | --- | --- | --- | --- | --- |
| MACCS | RF | 0.80±0.02 | 0.86±0.01 | 0.77±0.02 | 0.74±0.03 | 0.85±0.03 | 0.81±0.02 |
|  | SVM | 0.78±0.01 | 0.86±0.01 | 0.74±0.02 | 0.69±0.05 | 0.88±0.05 | 0.80±0.02 |
|  | kNN | 0.75±0.02 | 0.81±0.02 | 0.69±0.02 | 0.59±0.04 | 0.90±0.02 | 0.78±0.01 |
| Morgan | RF | 0.78±0.02 | 0.85±0.01 | 0.74±0.02 | 0.68±0.05 | 0.87±0.06 | 0.80±0.02 |
|  | SVM | 0.79±0.02 | 0.87±0.02 | 0.75±0.02 | 0.72±0.04 | 0.86±0.06 | 0.80±0.03 |
|  | kNN | 0.61±0.02 | 0.68±0.02 | 0.56±0.01 | 0.26±0.04 | 0.95±0.03 | 0.71±0.02 |
| Mordred | RF | 0.81±0.02 | 0.87±0.02 | 0.78±0.02 | 0.76±0.02 | 0.86±0.04 | 0.82±0.02 |
|  | SVM | 0.82±0.03 | 0.89±0.02 | 0.79±0.04 | 0.77±0.06 | 0.87±0.03 | 0.83±0.02 |
|  | kNN | 0.78±0.04 | 0.86±0.02 | 0.73±0.04 | 0.67±0.06 | 0.89±0.04 | 0.81±0.03 |
| Concat. Vectors | **FCNN** | **0.82±0.01** | **0.88±0.01** | **0.78±0.03** | **0.75±0.05** | **0.88±0.04** | **0.83±0.01** |

**Table S4.** 5-fold cross validation performance comparison of acute dermal toxicity prediction using different featurization methods

| Feature | Model | Acc | AUC | Pre | Spe | Sen | F1 |
| --- | --- | --- | --- | --- | --- | --- | --- |
| MACCS | RF | 0.83±0.02 | 0.90±0.02 | 0.83±0.03 | 0.83±0.04 | 0.83±0.02 | 0.83±0.02 |
|  | SVM | 0.83±0.02 | 0.90±0.02 | 0.81±0.03 | 0.80±0.03 | 0.86±0.02 | 0.84±0.02 |
|  | kNN | 0.80±0.03 | 0.88±0.02 | 0.75±0.03 | 0.71±0.05 | 0.89±0.03 | 0.81±0.02 |
| Morgan | RF | 0.81±0.03 | 0.89±0.02 | 0.79±0.03 | 0.78±0.04 | 0.84±0.03 | 0.82±0.03 |
|  | SVM | 0.82±0.02 | 0.90±0.02 | 0.81±0.03 | 0.80±0.04 | 0.84±0.01 | 0.82±0.02 |
|  | kNN | 0.67±0.03 | 0.77±0.04 | 0.61±0.03 | 0.38±0.07 | 0.96±0.02 | 0.74±0.02 |
| Mordred | RF | 0.82±0.01 | 0.90±0.03 | 0.82±0.02 | 0.81±0.03 | 0.83±0.01 | 0.82±0.01 |
|  | SVM | 0.82±0.02 | 0.90±0.03 | 0.84±0.04 | 0.84±0.04 | 0.80±0.03 | 0.82±0.02 |
|  | kNN | 0.81±0.03 | 0.90±0.04 | 0.81±0.04 | 0.80±0.04 | 0.86±0.04 | 0.83±0.03 |
| Concat. Vectors | **FCNN** | **0.85±0.02** | **0.92±0.02** | **0.85±0.03** | **0.85±0.03** | **0.85±0.02** | **0.85±0.02** |

# **Section 4:** QMRF for skin sensitization model

|  | **Element** | **Explanation** |
| --- | --- | --- |
| **1.** | **QSAR identifier** |  |
| 1.1. | QSAR identifier (title) | QSPR model for the prediction of skin sensitization of molecules based on their structural characteristics |
| 1.2 | Other related models | J. V. B. Borba et al., “STopTox: An in Silico Alternative to Animal Testing for Acute Systemic and Topical Toxicity,” Environ. Health Perspect., vol. 130, no. 2, p. 027012, Feb. 2022, doi: 10.1289/EHP9341 |
| 1.3. | Software coding the model | Python v. 3.10  Tensorflow v. 2.16.1  RDKit v. 2024.09.6 |
| **2.** | **General information** |  |
| 2.0 | Abstract | Within this web-tool, users can upload one or several compounds of interest and acquire a prediction of their skin sensitization potential. The platform provides three different options for inserting the required input to the model: 1. The user can draw the chemical structure of interest using the drawing tool, 2. The user can enter the SMILES notation of one or several compounds separated by newlines and, 3. The user can select and import an SDF file with several compounds. |
| 2.1. | Date of QMRF | 11 April 2025 |
| 2.2. | QMRF author(s) and contact details | Nikoletta-Maria Koutroumpa ([koutroumpa@novamechanics.com](mailto:koutroumpa@novamechanics.com))  Dimitra-Danai Varsou ([varsou@novamechanics.com](mailto:varsou@novamechanics.com))  Maria Antoniou ([antoniou@novamechanics.com](mailto:antoniou@novamechanics.com))  Eleni Papadopoulou ([papadopoulou@novamechanics.com](mailto:papadopoulou@novamechanics.com))  Antreas Afantitis ([afantitis@novamechanics.com](mailto:afantitis@novamechanics.com)) |
| 2.3. | Date of QMRF update(s) | NA |
| 2.4. | QMRF update(s) | NA |
| 2.5. | Model developer(s) and contact details | Nikoletta-Maria Koutroumpa ([koutroumpa@novamechanics.com](mailto:koutroumpa@novamechanics.com))  Dimitra-Danai Varsou ([varsou@novamechanics.com](mailto:varsou@novamechanics.com))  Maria Antoniou ([antoniou@novamechanics.com](mailto:antoniou@novamechanics.com))  Eleni Papadopoulou ([papadopoulou@novamechanics.com](mailto:papadopoulou@novamechanics.com))  Antreas Afantitis ([afantitis@novamechanics.com](mailto:afantitis@novamechanics.com)) |
| 2.6. | Date of model development and/or publication | 15 February 2025 |
| 2.7. | Reference(s) to main scientific papers and/or software package | Pending publication |
| 2.8. | Availability of information about the model | The model is proprietary: the source code is confidential; however, the description of the modelling workflow is presented in the original research article, training and validation sets are available as supplementary information of the original research article and the model is implemented as a public web service. |
| 2.9. | Availability of another QMRF for exactly the same model | NA |
| **3** | **Defining the endpoint - OECD Principle 1: “A DEFINED ENDPOINT"** | **PRINCIPLE 1: “A DEFINED ENDPOINT". ENDPOINT refers to any physicochemical, biological, or environmental property/activity/effect that can be measured and therefore modelled. The intent of PRINCIPLE 1 (a (Q)SAR should be associated with a defined endpoint) is to ensure clarity in the endpoint being predicted by a given model, since a given endpoint could be determined by different experimental protocols and under different experimental conditions. It is therefore important to identify the experimental system and test conditions that is being modelled by the Q)SAR.** |
| 3.1. | Species | Local lymph node assay (LLNA) |
| 3.2. | Endpoint | Skin sensitization chemical records with LLNA data |
| 3.3 | Comment on endpoint | Skin sensitization is the most common immune response experienced in humans. It occures when a person is exposed to sensitizing chemicals, which may lead to allergic contact fermatitis. Evaluating the skin sensitization potential is essential for safety assessment of chemicals. |
| 3.4. | Endpoint units | NA |
| 3.5. | Dependent variable | Skin sensitization data is a categorical endpoint: “sensitizer” labels indicate potentially skin sensitizing chemicals, and “non-sensitizer” labels indicate chemicals not causing skin sensitization. |
| 3.6. | Experimental protocol | Skin sensitization data were collected from the National Toxicology Program Interagency Center for the Evaluation of Alternative Toxicological Methods on behalf of ICCVAM [1] and the Registration, Evaluation, Authorization and restriction of Chemicals (REACH) Study Results Database [2].  [1] ICCVAM (Interagency Coordinating Committee on the Validation of Alternative and Methods), NICEATM Murine Local Lymph Node Assay (LLNA) Database, 2013. Accessed: Feb. 02, 2025. [Online]. Available: [https://ntp.niehs.nih.gov/whatwestudy/niceatm/test-method-evaluations/skin- sens/llna/index.html](https://ntp.niehs.nih.gov/whatwestudy/niceatm/test-method-evaluations/skin-%20sens/llna/index.html)  [2] ECHA (European Chemical Agency), OECD (Organization for Economic Co- and operation and Development)., REACH Study Results - IUCLID, 2019. Accessed: Feb. 02, 2025. [Online]. Available: https://iuclid6.echa.europa.eu/reach-study-results |
| 3.7. | Endpoint data quality and variability | Complete dataset:   \| Sensitizers \| Non-sensitizers \| Sensitizers / non-sensitizers \| \| --- \| --- \| --- \| \| 481 \| 519 \| 0.93 \|   . |
| **4** | **Defining the algorithm - OECD Principle 2 : “AN UNAMBIGUOUS ALGORITHM”** | **PRINCIPLE 2: “AN UNAMBIGUOUS ALGORITHM”. The (Q)SAR estimate of an endpoint is the result of applying an ALGORITHM to a set of structural parameters which describe the chemical structure. The intent of PRINCIPLE 2 (a (Q)SAR should be associated with an unambiguous algorithm) is to ensure transparency in the model algorithm that generates predictions of an endpoint from information on chemical structure and/or physicochemical properties. In this context, algorithm refers to any mathematical equation, decision rule or output approach.** |
| 4.1. | Type of model | Fully connected neural network |
| 4.2. | Explicit algorithm | Learning rate: 1e-3  Batch size: 32  Number of nodes of first dense layer: 1024  Number of nodes of second dense layer: 256  Dropout rate for the first dropout layer after the first dense layer: 0.3  Dropout rate for the second dropout layer after the second dense layer: 0.1 |
| 4.3. | Descriptors in the model | The compounds were represented as Morgan fingerprints, MACCS keys, and Mordred descriptors. |
| 4.4. | Descriptor selection | The Morgan fingerprints and MACCS keys were used as bit vectors to represent the model.  A combination of univariate and multivariate selection methods was then applied to pinpoint the most informative Mordred descriptors. First, an Analysis of Variance (ANOVA) was performed to identify descriptors with statistically significant differences between classes (e.g., sensitizers vs. non-sensitizers). Subsequently, L1-penalized linear regression (also known as Lasso regression) was used to further reduce descriptor complexity by imposing sparsity, effectively zeroing out less relevant coefficients and producing a concise set of Mordred descriptors. |
| 4.5. | Algorithm and descriptor generation | RDKit: We utilized RDKit Open-Source Toolkit for Cheminformatics for MACCS keys and Morgan Fingerprints generation [1]  [1] RDKit, RDKit: Open-source cheminformatics. Accessed: May 02, 2025. [Online]. Available: <https://www.rdkit.org/>  Mordred: We utilized Mordred package for molecular descriptor calculation. Mordred descriptors [2] constitute a set of more than 1,800 two- and three-dimensional descriptors that capture geometric, electronic, topological, and hybrid characteristics. In this study, only the two-dimensional Mordred descriptors were utilized to ensure consistency with other molecular representations.  [2] H. Moriwaki, Y.-S. Tian, N. Kawashita, and T. Takagi, “Mordred: a molecular descriptor calculator,” J. Cheminformatics, vol. 10, no. 1, p. 4, Dec. 2018, doi: 10.1186/s13321-018-0258-y |
| 4.6. | Software name and version for descriptor generation | RDKit v. 2024.09.6  Mordred v. 2.0.6 |
| 4.7. | Chemicals/Descriptors ratio | Our dataset contains 1000 chemicals. The model uses a combined input feature space of 2278 descriptors, comprising of 2048-bit Morgan fingerprint vectors, 167-bit MACCS keys vectors and 63 Mordred descriptors. While the ratio of chemicals/descriptors should be high in classical QSPR modeling (due to risk of overfitting when the number of descriptors exceeds the number of chemicals), this is not a limiting factor in deep learning approaches. Fully Connected Neural Networks handle high-dimensional input spaces by learning complex representations. The number of hidden neurons in the second layer 256 which then pass through the output layer for the final classification of the compounds. |
| **5** | **Defining the applicability domain - OECD Principle 3: “A DEFINED DOMAIN OF APPLICABILITY”** | **PRINCIPLE 3: “A DEFINED DOMAIN OF APPLICABILITY”. APPLICABILITY DOMAIN refers to the response and chemical structure space in which the model makes predictions with a given reliability. Ideally the applicability domain should express the structural, physicochemical and response space of the model. The CHEMICAL STRUCTURE (x variable) space can be expressed by information on physicochemical properties and/or structural fragments. The RESPONSE (y variable) can be any physicochemical, biological or environmental effect that is being predicted. According to PRINCIPLE 3 a (Q)SAR should be associated with a defined domain of applicability. Section 5 can be repeated (e.g., 5.a, 5.b, 5.c, etc) as many times as necessary if more than one method has been used to assess the applicability domain.** |
| 5.1. | Description of the applicability domain of the model | In this study, two approaches were used: a Euclidean distance-based method for Mordred descriptor features and a Tanimoto similarity-based method for MACCS keys and Morgan fingerprints.  For Euclidean distance-based method, the Euclidean distance between the test compound and the centroid in training data is calculated and if this value is larger than the calculated APD threshold, the test compound is considered outside the applicability domain.  For similarity-based method, if the average Tanimoto similarity between the test compound and its k most similar compounds in the training exceed the APD threshold calculated on the training compounds, then is considered to be in the applicability domain.  For the models trained on concatenated features -combining Mordred descriptors and fingerprints- we considered both approaches independently to evaluate their individual contribution to the AD. Specifically, we applied the Euclidean distance-based approach to the descriptor subset and the similarity-based approach to the fingerprint subset, both MACCS and Morgan fingerprints. To determine whether a compound falls within the AD of the model, we adopted a consensus approach, considering a compound inside the AD if it meets at least two of the three defined thresholds. |
| 5.2. | Method used to assess the applicability domain | For the Euclidean distance-based AD, the centroid of the training compounds was calculated as the mean of each scaled descriptor. The distances between all training compounds and the centroid were calculated, and the largest distance was adopted as the threshold $d_{t}$. Then, the Euclidean distance $d_{Euc,i}$ between the test compound $i$ and the centroid was calculated according to:  $d_{Euc,i}=\sqrt{\sum\left( x_{j,i}-x_{j, centroid} \right)^{2}}$  where $x_{j,i}$is the $j^{th}$ scaled descriptor of compound $i$ and $x_{j,centroid}$ is the $j^{th}$ scaled descriptor of the centroid. In case the $d_{Euc,i}$ of compound $i$ is larger than the $d_{t}$, the compound is considered outside the AD.  The similarity-based AD was adopted for the fingerprint representation of compounds. This methodology computes the similarity matrix between the training compounds and the test compounds. A similarity threshold is calculated as defined:  $S_{T}=\bar{\gamma}+Z\sigma$  where $\bar{\gamma}$ represents the average Tanimoto similarity between training compounds, $\sigma$ is the standard deviation of the computed Tanimoto similarities, and $Z$ is a predefined value indicating the significance level. If the average Tanimoto similarity between the test compound and its $k$ most similar compounds in the training set exceeds the threshold $S_{T}$, it is consided to be in the AD, otherwise the compound is considered outside the AD. |
| 5.3. | Software name and version for applicability domain assessment | Python v. 3.10 |
| 5.4. | Limits of applicability | Euclidean-based APD threshold (Mordred descriptors): 38.193  Similarity-based APD threshold (MACCS keys): 0.332  Similarity-based APD threshold (Morgan Fingerprints): 0.148 |
| **6** | **Defining goodness-of-fit and robustness (internal validation) – OECD Principle 4: “APPROPRIATE MEASURES OF GOODNESS-OF-FIT, ROBUSTENESS AND PREDICTIVITY”** | **PRINCIPLE 4: “APPROPRIATE MEASURES OF GOODNESS-OF-FIT, ROBUSTENESS AND PREDICTIVITY”. PRINCIPLE 4 expresses the need to perform validation to establish the performance of the model. GOODNESS-OF-FIT and ROBUSTNESS refer to the internal model performance.** |
| 6.1. | Availability of the training set | Available at the ChemPharos database:  https://db.chempharos.eu/datasets/Datasets.zul |
| 6.2. | Available information for the training set | a) Chemical names (common names and/or IUPAC names); b) CAS numbers; c) SMILES; d) InChI codes; e) Structural formula; f) MW |
| 6.3. | Data for each descriptor variable for the training set | Available at the ChemPharos database:  [https://db.chempharos.eu/datasets/Datasets.zul](https://db.chempharos.eu/datasets/Datasets.zul?datasetID=ds11) |
| 6.4. | Data for the dependent variable for the training set | Available at the ChemPharos database:  [https://db.chempharos.eu/datasets/Datasets.zul](https://db.chempharos.eu/datasets/Datasets.zul?datasetID=ds11) |
| 6.5. | Other information about the training set | Random splitting (stratified based on the chemicals’ class) was used for partitioning: 800 out of 1000 molecules were included in the training set for model development. From the remaining 200 molecules, 100 were randomly selected as the validation set for hyperparameter optimization and the other 100 molecules were used as a test set for evaluating model performance. |
| 6.6. | Pre-processing of data before modelling | Checking SMILES structures with RDKit molecular object.  Removal of columns containing NA values.  In case of unbalanced dataset, an undersampling strategy was employed to result in a balanced dataset.  Z-score normalization of descriptors.  Variable selection of Mordred descriptors was performed according to §4.4. |
| 6.7. | Statistics for goodness-of-fit | Training set:   \| **Metric** \| **Value** \| \| --- \| --- \| \| ACC \| 0.981 \| \| AUC \| 0.997 \| \| PRE \| 0.967 \| \| SPE \| 0.969 \| \| SEN \| 0.995 \| \| MCC \| 0.963 \| \| F1 \| 0.981 \| |
| 6.8. | Robustness - Statistics obtained by leave-one-out cross-validation | NA |
| 6.9. | Robustness - Statistics obtained by leave-many-out cross-validation | Robustness – Statistics obtained by 5-fold cross-validation (random splitting of data).   \| **Metric** \| **Value** \| \| --- \| --- \| \| ACC \| 0.721 \| \| AUC \| 0.792 \| \| PRE \| 0.699 \| \| SPE \| 0.704 \| \| SEN \| 0.739 \| \| MCC \| 0.444 \| \| F1 \| 0.718 \| |
| 6.10. | Robustness - Statistics obtained by Y-scrambling | \| **Iteration** \| **ACC** \| **AUC** \| **SEN** \| **SPE** \| **MCC** \| \| --- \| --- \| --- \| --- \| --- \| --- \| \| 1 \| 0.540 \| 0.527 \| 0.667 \| 0.423 \| 0.092 \| \| 2 \| 0.530 \| 0.515 \| 0.438 \| 0.615 \| 0.054 \| \| 3 \| 0.440 \| 0.445 \| 0.583 \| 0.308 \| -0.113 \| \| 4 \| 0.560 \| 0.595 \| 0.604 \| 0.519 \| 0.124 \| \| 5 \| 0.440 \| 0.465 \| 0.375 \| 0.500 \| -0.126 \| |
| 6.11. | Robustness - Statistics obtained by bootstrap | NA |
| 6.12. | Robustness - Statistics obtained by other methods | NA |
| **7** | **Defining predictivity (external validation) – OECD Principle 4: “APPROPRIATE MEASURES OF GOODNESS-OF-FIT, ROBUSTENESS AND PREDICTIVITY”** | **PRINCIPLE 4: “APPROPRIATE MEASURES OF GOODNESS-OF-FIT, ROBUSTENESS AND PREDICTIVITY”. PRINCIPLE 4 expresses the need to perform validation to establish the performance of the model. PREDICTIVITY refers to the external model validation. Section 7 can be repeated (e.g., 7.a, 7.b, 7.c, etc) as many times as necessary if more validation studies need to be reported in the QMRF.** |
| 7.1. | Availability of the external validation set | Available at the ChemPharos database:  https://db.chempharos.eu/datasets/Datasets.zul |
| 7.2. | Available information for the external validation set | a) Chemical names (common names and/or IUPAC names); b) CAS numbers; c) SMILES; d) InChI codes; e) Structural formula; f) MW |
| 7.3. | Data for each descriptor variable for the external validation set | Available at the ChemPharos database:  [https://db.chempharos.eu/datasets/Datasets.zul](https://db.chempharos.eu/datasets/Datasets.zul?datasetID=ds11) |
| 7.4. | Data for the dependent variable for the external validation set | Available at the ChemPharos database:  https://db.chempharos.eu/datasets/Datasets.zul |
| 7.5. | Other information about the external validation set | 100 molecules were included in the external validation (test set), which was not involved in model development, but it was rather used solely for validating purposes. |
| 7.6. | Experimental design of test set | Randomly selected from the first partitioning of data (200 molecules). See also §6.5. |
| 7.7. | Predictivity - Statistics obtained by external validation | \| **Metric** \| **Value** \| \| --- \| --- \| \| ACC \| 0.85 \| \| AUC \| 0.881 \| \| PRE \| 0.851 \| \| SPE \| 0.865 \| \| SEN \| 0.833 \| \| MCC \| 0.699 \| \| F1 \| 0.842 \| |
| 7.8. | Predictivity - Assessment of the external validation set | The external validation set (test set) is 10% of the initial dataset, 100% of predictions fall within the domain of applicability. |
| 7.9. | Comments on the external validation of the model | Test and validation sets were normalized based on the Z-score normalization applied on the training set. |
| **8** | **Providing a mechanistic interpretation - OECD Principle 5: “A MECHANISTIC INTERPRETATION, IF POSSIBLE”** | **PRINCIPLE 5: “A MECHANISTIC INTERPRETATION, IF POSSIBLE”. According to PRINCIPLE 5, a (Q)SAR should be associated with a mechanistic interpretation, if possible.** |
| 8.1. | Mechanistic basis of the model | NA |
| 8.2. | A priori or a posteriori mechanistic interpretation | NA |
| 8.3. | Other information about the mechanistic interpretation | NA |
| **9** | **Miscellaneous information** |  |
| 9.1. | Comments | The dataset was from Borba *et al*. [1], of 1000 chemicals from different sources.  [1] J. V. B. Borba et al., “STopTox: An in Silico Alternative to Animal Testing for Acute Systemic and Topical Toxicity,” Environ. Health Perspect., vol. 130, no. 2, p. 027012, Feb. 2022, doi: 10.1289/EHP9341 |
| 9.2. | Bibliography | 1. J. V. B. Borba et al., “STopTox: An in Silico Alternative to Animal Testing for Acute Systemic and Topical Toxicity,” Environ. Health Perspect., vol. 130, no. 2, p. 027012, Feb. 2022, doi: 10.1289/EHP9341 2. ICCVAM (Interagency Coordinating Committee on the Validation of Alternative and Methods), NICEATM Murine Local Lymph Node Assay (LLNA) Database, 2013. Accessed: Feb. 02, 2025. [Online]. Available: [https://ntp.niehs.nih.gov/whatwestudy/niceatm/test-method-evaluations/skin- sens/llna/index.html](https://ntp.niehs.nih.gov/whatwestudy/niceatm/test-method-evaluations/skin-%20sens/llna/index.html) 3. ECHA (European Chemical Agency), OECD (Organization for Economic Co- and operation and Development)., REACH Study Results - IUCLID, 2019. Accessed: Feb. 02, 2025. [Online]. Available: https://iuclid6.echa.europa.eu/reach-study-results |
| 9.3 | Supporting information | NA |

# **Section 5:** QMRF for skin irritation/corrosion model

|  | **Element** | **Explanation** |
| --- | --- | --- |
| **1.** | **QSAR identifier** |  |
| 1.1. | QSAR identifier (title) | QSPR model for the prediction of skin irritation and corrosion of molecules based on their structural characteristics |
| 1.2 | Other related models | J. V. B. Borba et al., “STopTox: An in Silico Alternative to Animal Testing for Acute Systemic and Topical Toxicity,” Environ. Health Perspect., vol. 130, no. 2, p. 027012, Feb. 2022, doi: 10.1289/EHP9341 |
| 1.3. | Software coding the model | Python v. 3.10  Tensorflow v. 2.16.1  RDKit v. 2024.09.6 |
| **2.** | **General information** |  |
| 2.0 | Abstract | Within this web-tool, users can upload one or several compounds of interest and acquire a prediction of their skin irritation and corrosion potential. The platform provides three different options for inserting the required input to the model: 1. The user can draw the chemical structure of interest using the drawing tool, 2. The user can enter the SMILES notation of one or several compounds separated by newlines and, 3. The user can select and import an SDF file with several compounds. |
| 2.1. | Date of QMRF | 11 April 2025 |
| 2.2. | QMRF author(s) and contact details | Nikoletta-Maria Koutroumpa ([koutroumpa@novamechanics.com](mailto:koutroumpa@novamechanics.com))  Dimitra-Danai Varsou ([varsou@novamechanics.com](mailto:varsou@novamechanics.com))  Maria Antoniou ([antoniou@novamechanics.com](mailto:antoniou@novamechanics.com))  Eleni Papadopoulou ([papadopoulou@novamechanics.com](mailto:papadopoulou@novamechanics.com))  Antreas Afantitis ([afantitis@novamechanics.com](mailto:afantitis@novamechanics.com)) |
| 2.3. | Date of QMRF update(s) | NA |
| 2.4. | QMRF update(s) | NA |
| 2.5. | Model developer(s) and contact details | Nikoletta-Maria Koutroumpa ([koutroumpa@novamechanics.com](mailto:koutroumpa@novamechanics.com))  Dimitra-Danai Varsou ([varsou@novamechanics.com](mailto:varsou@novamechanics.com))  Maria Antoniou ([antoniou@novamechanics.com](mailto:antoniou@novamechanics.com))  Eleni Papadopoulou ([papadopoulou@novamechanics.com](mailto:papadopoulou@novamechanics.com))  Antreas Afantitis ([afantitis@novamechanics.com](mailto:afantitis@novamechanics.com)) |
| 2.6. | Date of model development and/or publication | 15 February 2025 |
| 2.7. | Reference(s) to main scientific papers and/or software package | Pending publication |
| 2.8. | Availability of information about the model | The model is proprietary: the source code is confidential; however, the description of the modelling workflow is presented in the original research article, training and validation sets are available as supplementary information of the original research article and the model is implemented as a public web service. |
| 2.9. | Availability of another QMRF for exactly the same model | NA |
| **3** | **Defining the endpoint - OECD Principle 1: “A DEFINED ENDPOINT"** | **PRINCIPLE 1: “A DEFINED ENDPOINT". ENDPOINT refers to any physicochemical, biological, or environmental property/activity/effect that can be measured and therefore modelled. The intent of PRINCIPLE 1 (a (Q)SAR should be associated with a defined endpoint) is to ensure clarity in the endpoint being predicted by a given model, since a given endpoint could be determined by different experimental protocols and under different experimental conditions. It is therefore important to identify the experimental system and test conditions that is being modelled by the Q)SAR.** |
| 3.1. | Species | Albino rabbit |
| 3.2. | Endpoint | Skin irritation/corrosion chemical records |
| 3.3 | Comment on endpoint | Skin irritation is a significant adverse effect associated with chemicals and drug substances, which poses significant challenges in dermatology. |
| 3.4. | Endpoint units | NA |
| 3.5. | Dependent variable | Skin irritation/corrosion data is a categorical endpoint: “irritant” labels indicate potentially skin irritating chemicals, and “non-irritant” labels indicate chemicals not causing skin irritation. |
| 3.6. | Experimental protocol | Skin irritation/corrosion data were collected from the Registration, Evaluation, Authorization and restriction of Chemicals (REACH) Study Results Database [2].  [2] ECHA (European Chemical Agency), OECD (Organization for Economic Co- and operation and Development)., REACH Study Results - IUCLID, 2019. Accessed: Feb. 02, 2025. [Online]. Available: https://iuclid6.echa.europa.eu/reach-study-results |
| 3.7. | Endpoint data quality and variability | Complete dataset:   \| Irritants \| Non-irritants \| Irritants / non-irritants \| \| --- \| --- \| --- \| \| 317 \| 694 \| 0.46 \|   Balanced dataset:   \| Irritants \| Non-irritants \| Irritants / non-irritants \| \| --- \| --- \| --- \| \| 317 \| 317 \| 1.0 \| |
| **4** | **Defining the algorithm - OECD Principle 2 : “AN UNAMBIGUOUS ALGORITHM”** | **PRINCIPLE 2: “AN UNAMBIGUOUS ALGORITHM”. The (Q)SAR estimate of an endpoint is the result of applying an ALGORITHM to a set of structural parameters which describe the chemical structure. The intent of PRINCIPLE 2 (a (Q)SAR should be associated with an unambiguous algorithm) is to ensure transparency in the model algorithm that generates predictions of an endpoint from information on chemical structure and/or physicochemical properties. In this context, algorithm refers to any mathematical equation, decision rule or output approach.** |
| 4.1. | Type of model | Fully connected neural network |
| 4.2. | Explicit algorithm | Learning rate: 1e-4  Batch size: 32  Number of nodes of first dense layer: 512  Number of nodes of second dense layer: 256  Dropout rate for the first dropout layer after the first dense layer: 0.3  Dropout rate for the second dropout layer after the second dense layer: 0.1 |
| 4.3. | Descriptors in the model | The compounds were represented as Morgan fingerprints, MACCS keys, and Mordred descriptors. |
| 4.4. | Descriptor selection | The Morgan fingerprints and MACCS keys were used as bit vectors to represent the model.  A combination of univariate and multivariate selection methods was then applied to pinpoint the most informative Mordred descriptors. First, an Analysis of Variance (ANOVA) was performed to identify descriptors with statistically significant differences between classes (e.g., irritants vs. non-irritants). Subsequently, L1-penalized linear regression (also known as Lasso regression) was used to further reduce descriptor complexity by imposing sparsity, effectively zeroing out less relevant coefficients and producing a concise set of Mordred descriptors. |
| 4.5. | Algorithm and descriptor generation | RDKit: We utilized RDKit Open-Source Toolkit for Cheminformatics for MACCS keys and Morgan Fingerprints generation [1]  [1] RDKit, RDKit: Open-source cheminformatics. Accessed: May 02, 2025. [Online]. Available: <https://www.rdkit.org/>  Mordred: We utilized Mordred package for molecular descriptor calculation. Mordred descriptors [2] constitute a set of more than 1,800 two- and three-dimensional descriptors that capture geometric, electronic, topological, and hybrid characteristics. In this study, only the two-dimensional Mordred descriptors were utilized to ensure consistency with other molecular representations.  [2] H. Moriwaki, Y.-S. Tian, N. Kawashita, and T. Takagi, “Mordred: a molecular descriptor calculator,” J. Cheminformatics, vol. 10, no. 1, p. 4, Dec. 2018, doi: 10.1186/s13321-018-0258-y |
| 4.6. | Software name and version for descriptor generation | RDKit v. 2024.09.6  Mordred v. 2.0.6 |
| 4.7. | Chemicals/Descriptors ratio | Our dataset contains 634 chemicals. The model uses a combined input feature space of 2278 descriptors, comprising of 2048-bit Morgan fingerprint vectors, 167-bit MACCS keys vectors and 62 Mordred descriptors. While the ratio of chemicals/descriptors should be high in classical QSPR modeling (due to risk of overfitting when the number of descriptors exceeds the number of chemicals), this is not a limiting factor in deep learning approaches. Fully Connected Neural Networks handle high-dimensional input spaces by learning complex representations. The number of hidden neurons in the second layer 256 which then pass through the output layer for the final classification of the compounds. |
| **5** | **Defining the applicability domain - OECD Principle 3: “A DEFINED DOMAIN OF APPLICABILITY”** | **PRINCIPLE 3: “A DEFINED DOMAIN OF APPLICABILITY”. APPLICABILITY DOMAIN refers to the response and chemical structure space in which the model makes predictions with a given reliability. Ideally the applicability domain should express the structural, physicochemical and response space of the model. The CHEMICAL STRUCTURE (x variable) space can be expressed by information on physicochemical properties and/or structural fragments. The RESPONSE (y variable) can be any physicochemical, biological or environmental effect that is being predicted. According to PRINCIPLE 3 a (Q)SAR should be associated with a defined domain of applicability. Section 5 can be repeated (e.g., 5.a, 5.b, 5.c, etc) as many times as necessary if more than one method has been used to assess the applicability domain.** |
| 5.1. | Description of the applicability domain of the model | In this study, two approaches were used: a Euclidean distance-based method for Mordred descriptor features and a Tanimoto similarity-based method for MACCS keys and Morgan fingerprints.  For Euclidean distance-based method, the Euclidean distance between the test compound and the centroid in training data is calculated and if this value is larger than the calculated APD threshold, the test compound is considered outside the applicability domain.  For similarity-based method, if the average Tanimoto similarity between the test compound and its k most similar compounds in the training exceed the APD threshold calculated on the training compounds, then is considered to be in the applicability domain.  For the models trained on concatenated features -combining Mordred descriptors and fingerprints- we considered both approaches independently to evaluate their individual contribution to the AD. Specifically, we applied the Euclidean distance-based approach to the descriptor subset and the similarity-based approach to the fingerprint subset, both MACCS and Morgan fingerprints. To determine whether a compound falls within the AD of the model, we adopted a consensus approach, considering a compound inside the AD if it meets at least two of the three defined thresholds. |
| 5.2. | Method used to assess the applicability domain | For the Euclidean distance-based AD, the centroid of the training compounds was calculated as the mean of each scaled descriptor. The distances between all training compounds and the centroid were calculated, and the largest distance was adopted as the threshold $d_{t}$. Then, the Euclidean distance $d_{Euc,i}$ between the test compound $i$ and the centroid was calculated according to:  $d_{Euc,i}=\sqrt{\sum\left( x_{j,i}-x_{j, centroid} \right)^{2}}$  where $x_{j,i}$is the $j^{th}$ scaled descriptor of compound $i$ and $x_{j,centroid}$ is the $j^{th}$ scaled descriptor of the centroid. In case the $d_{Euc,i}$ of compound $i$ is larger than the $d_{t}$, the compound is considered outside the AD.  The similarity-based AD was adopted for the fingerprint representation of compounds. This methodology computes the similarity matrix between the training compounds and the test compounds. A similarity threshold is calculated as defined:  $S_{T}=\bar{\gamma}+Z\sigma$  where $\bar{\gamma}$ represents the average Tanimoto similarity between training compounds, $\sigma$ is the standard deviation of the computed Tanimoto similarities, and $Z$ is a predefined value indicating the significance level. If the average Tanimoto similarity between the test compound and its $k$ most similar compounds in the training set exceeds the threshold $S_{T}$, it is consided to be in the AD, otherwise the compound is considered outside the AD. |
| 5.3. | Software name and version for applicability domain assessment | Python v. 3.10 |
| 5.4. | Limits of applicability | Euclidean-based APD threshold (Mordred descriptors): 21.188  Similarity-based APD threshold (MACCS keys): 0.344  Similarity-based APD threshold (Morgan Fingerprints): 0.149 |
| **6** | **Defining goodness-of-fit and robustness (internal validation) – OECD Principle 4: “APPROPRIATE MEASURES OF GOODNESS-OF-FIT, ROBUSTENESS AND PREDICTIVITY”** | **PRINCIPLE 4: “APPROPRIATE MEASURES OF GOODNESS-OF-FIT, ROBUSTENESS AND PREDICTIVITY”. PRINCIPLE 4 expresses the need to perform validation to establish the performance of the model. GOODNESS-OF-FIT and ROBUSTNESS refer to the internal model performance.** |
| 6.1. | Availability of the training set | Available at the ChemPharos database:  https://db.chempharos.eu/datasets/Datasets.zul |
| 6.2. | Available information for the training set | a) Chemical names (common names and/or IUPAC names); b) CAS numbers; c) SMILES; d) InChI codes; e) Structural formula; f) MW |
| 6.3. | Data for each descriptor variable for the training set | Available at the ChemPharos database:  https://db.chempharos.eu/datasets/Datasets.zul |
| 6.4. | Data for the dependent variable for the training set | Available at the ChemPharos database:  https://db.chempharos.eu/datasets/Datasets.zul |
| 6.5. | Other information about the training set | Random splitting (stratified based on the chemicals’ class) was used for partitioning: 507 out of 634 molecules were included in the training set for model development. From the remaining 127 molecules, 63 were randomly selected as the validation set for hyperparameter optimization and the other 64 molecules were used as a test set for evaluating model performance. |
| 6.6. | Pre-processing of data before modelling | Checking SMILES structures with RDKit molecular object.  Removal of columns containing NA values.  In case of unbalanced dataset, an undersampling strategy was employed to result in a balanced dataset.  Z-score normalization of descriptors.  Variable selection of Mordred descriptors was performed according to §4.4. |
| 6.7. | Statistics for goodness-of-fit | Training set:   \| **Metric** \| **Value** \| \| --- \| --- \| \| ACC \| 0.947 \| \| AUC \| 0.991 \| \| PRE \| 0.909 \| \| SPE \| 0.902 \| \| SEN \| 0.992 \| \| MCC \| 0.897 \| \| F1 \| 0.949 \| |
| 6.8. | Robustness - Statistics obtained by leave-one-out cross-validation | NA |
| 6.9. | Robustness - Statistics obtained by leave-many-out cross-validation | Robustness – Statistics obtained by 5-fold cross-validation (random splitting of data).   \| **Metric** \| **Value** \| \| --- \| --- \| \| ACC \| 0.817 \| \| AUC \| 0.883 \| \| PRE \| 0.784 \| \| SPE \| 0.754 \| \| SEN \| 0.754 \| \| MCC \| 0.643 \| \| F1 \| 0.828 \| |
| 6.10. | Robustness - Statistics obtained by Y-scrambling | \| **Iteration** \| **ACC** \| **AUC** \| **SEN** \| **SPE** \| **MCC** \| \| --- \| --- \| --- \| --- \| --- \| --- \| \| 1 \| 0.509 \| 0.514 \| 0.619 \| 0.400 \| 0.124 \| \| 2 \| 0.484 \| 0.510 \| 0.281 \| 0.688 \| -0.034 \| \| 3 \| 0.516 \| 0.491 \| 0.250 \| 0.781 \| 0.037 \| \| 4 \| 0.500 \| 0.494 \| 0.313 \| 0.688 \| 0.000 \| \| 5 \| 0.469 \| 0.426 \| 0.469 \| 0.469 \| -0.063 \| |
| 6.11. | Robustness - Statistics obtained by bootstrap | NA |
| 6.12. | Robustness - Statistics obtained by other methods | NA |
| **7** | **Defining predictivity (external validation) – OECD Principle 4: “APPROPRIATE MEASURES OF GOODNESS-OF-FIT, ROBUSTENESS AND PREDICTIVITY”** | **PRINCIPLE 4: “APPROPRIATE MEASURES OF GOODNESS-OF-FIT, ROBUSTENESS AND PREDICTIVITY”. PRINCIPLE 4 expresses the need to perform validation to establish the performance of the model. PREDICTIVITY refers to the external model validation. Section 7 can be repeated (e.g., 7.a, 7.b, 7.c, etc) as many times as necessary if more validation studies need to be reported in the QMRF.** |
| 7.1. | Availability of the external validation set | Available at the ChemPharos database:  https://db.chempharos.eu/datasets/Datasets.zul |
| 7.2. | Available information for the external validation set | a) Chemical names (common names and/or IUPAC names); b) CAS numbers; c) SMILES; d) InChI codes; e) Structural formula; f) MW |
| 7.3. | Data for each descriptor variable for the external validation set | Available at the ChemPharos database:  https://db.chempharos.eu/datasets/Datasets.zul |
| 7.4. | Data for the dependent variable for the external validation set | Available at the ChemPharos database:  https://db.chempharos.eu/datasets/Datasets.zul |
| 7.5. | Other information about the external validation set | 64 molecules were included in the external validation (test set), which was not involved in model development, but it was rather used solely for validating purposes. |
| 7.6. | Experimental design of test set | Randomly selected from the first partitioning of data (127 molecules). See also §6.5. |
| 7.7. | Predictivity - Statistics obtained by external validation | \| **Metric** \| **Value** \| \| --- \| --- \| \| ACC \| 0.859 \| \| AUC \| 0.905 \| \| PRE \| 0.871 \| \| SPE \| 0.875 \| \| SEN \| 0.844 \| \| MCC \| 0.719 \| \| F1 \| 0.857 \| |
| 7.8. | Predictivity - Assessment of the external validation set | The external validation set (test set) is 10% of the initial dataset, 100% of predictions fall within the domain of applicability. |
| 7.9. | Comments on the external validation of the model | Test and validation sets were normalized based on the Z-score normalization applied on the training set. |
| **8** | **Providing a mechanistic interpretation - OECD Principle 5: “A MECHANISTIC INTERPRETATION, IF POSSIBLE”** | **PRINCIPLE 5: “A MECHANISTIC INTERPRETATION, IF POSSIBLE”. According to PRINCIPLE 5, a (Q)SAR should be associated with a mechanistic interpretation, if possible.** |
| 8.1. | Mechanistic basis of the model | NA |
| 8.2. | A priori or a posteriori mechanistic interpretation | NA |
| 8.3. | Other information about the mechanistic interpretation | NA |
| **9** | **Miscellaneous information** |  |
| 9.1. | Comments | The dataset was from Borba *et al*. [1], of 1011 chemicals from different sources.  [1] J. V. B. Borba et al., “STopTox: An in Silico Alternative to Animal Testing for Acute Systemic and Topical Toxicity,” Environ. Health Perspect., vol. 130, no. 2, p. 027012, Feb. 2022, doi: 10.1289/EHP9341 |
| 9.2. | Bibliography | 1. J. V. B. Borba et al., “STopTox: An in Silico Alternative to Animal Testing for Acute Systemic and Topical Toxicity,” Environ. Health Perspect., vol. 130, no. 2, p. 027012, Feb. 2022, doi: 10.1289/EHP9341 2. ECHA (European Chemical Agency), OECD (Organization for Economic Co- and operation and Development)., REACH Study Results - IUCLID, 2019. Accessed: Feb. 02, 2025. [Online]. Available: https://iuclid6.echa.europa.eu/reach-study-results |
| 9.3 | Supporting information | NA |

# **Section 6:** QMRF for acute dermal toxicity model

|  | **Element** | **Explanation** |
| --- | --- | --- |
| **1.** | **QSAR identifier** |  |
| 1.1. | QSAR identifier (title) | QSPR model for the prediction of acute dermal toxicity of molecules based on their structural characteristics |
| 1.2 | Other related models | J. V. B. Borba et al., “STopTox: An in Silico Alternative to Animal Testing for Acute Systemic and Topical Toxicity,” Environ. Health Perspect., vol. 130, no. 2, p. 027012, Feb. 2022, doi: 10.1289/EHP9341  R. Judson, “ToxValDB: Compiling Publicly Available In Vivo Toxicity Data,” 2019, The United States Environmental Protection Agency’s Center for Computational Toxicology and Exposure. doi: 10.23645/EPACOMPTOX.7800653 |
| 1.3. | Software coding the model | Python v. 3.10  Tensorflow v. 2.16.1  RDKit v. 2024.09.6 |
| **2.** | **General information** |  |
| 2.0 | Abstract | Within this web-tool, users can upload one or several compounds of interest and acquire a prediction of their acute dermal toxicity potential. The platform provides three different options for inserting the required input to the model: 1. The user can draw the chemical structure of interest using the drawing tool, 2. The user can enter the SMILES notation of one or several compounds separated by newlines and, 3. The user can select and import an SDF file with several compounds. |
| 2.1. | Date of QMRF | 11 April 2025 |
| 2.2. | QMRF author(s) and contact details | Nikoletta-Maria Koutroumpa ([koutroumpa@novamechanics.com](mailto:koutroumpa@novamechanics.com))  Dimitra-Danai Varsou ([varsou@novamechanics.com](mailto:varsou@novamechanics.com))  Maria Antoniou ([antoniou@novamechanics.com](mailto:antoniou@novamechanics.com))  Eleni Papadopoulou ([papadopoulou@novamechanics.com](mailto:papadopoulou@novamechanics.com))  Antreas Afantitis ([afantitis@novamechanics.com](mailto:afantitis@novamechanics.com)) |
| 2.3. | Date of QMRF update(s) | NA |
| 2.4. | QMRF update(s) | NA |
| 2.5. | Model developer(s) and contact details | Nikoletta-Maria Koutroumpa ([koutroumpa@novamechanics.com](mailto:koutroumpa@novamechanics.com))  Dimitra-Danai Varsou ([varsou@novamechanics.com](mailto:varsou@novamechanics.com))  Maria Antoniou ([antoniou@novamechanics.com](mailto:antoniou@novamechanics.com))  Eleni Papadopoulou ([papadopoulou@novamechanics.com](mailto:papadopoulou@novamechanics.com))  Antreas Afantitis ([afantitis@novamechanics.com](mailto:afantitis@novamechanics.com)) |
| 2.6. | Date of model development and/or publication | 15 February 2025 |
| 2.7. | Reference(s) to main scientific papers and/or software package | Pending publication |
| 2.8. | Availability of information about the model | The model is proprietary: the source code is confidential; however, the description of the modelling workflow is presented in the original research article, training and validation sets are available as supplementary information of the original research article and the model is implemented as a public web service. |
| 2.9. | Availability of another QMRF for exactly the same model | NA |
| **3** | **Defining the endpoint - OECD Principle 1: “A DEFINED ENDPOINT"** | **PRINCIPLE 1: “A DEFINED ENDPOINT". ENDPOINT refers to any physicochemical, biological, or environmental property/activity/effect that can be measured and therefore modelled. The intent of PRINCIPLE 1 (a (Q)SAR should be associated with a defined endpoint) is to ensure clarity in the endpoint being predicted by a given model, since a given endpoint could be determined by different experimental protocols and under different experimental conditions. It is therefore important to identify the experimental system and test conditions that is being modelled by the Q)SAR.** |
| 3.1. | Species | In vivo toxicity data from a diverse array of species including mammals (rats and mice) and fish. |
| 3.2. | Endpoint | Acute dermal toxicity chemical records |
| 3.3 | Comment on endpoint | Acute dermal toxicity is an adverse effect caused by a test chemical following a single uninterrupted expose by dermal application over a short period of time. |
| 3.4. | Endpoint units | NA |
| 3.5. | Dependent variable | Acute dermal toxicity data is a categorical endpoint: “toxic” labels indicate potentially acute dermal toxic chemicals, and “non-toxic” labels indicate chemicals not causing acute dermal toxicity. |
| 3.6. | Experimental protocol | Acute dermal toxicity data were collected from the Registration, Evaluation, Authorization and restriction of Chemicals (REACH) Study Results Database [1], the ToxValDB [2], and the literature [3].  [1] ECHA (European Chemical Agency), OECD (Organization for Economic Co- and operation and Development)., REACH Study Results - IUCLID, 2019. Accessed: Feb. 02, 2025. [Online]. Available: <https://iuclid6.echa.europa.eu/reach-study-results>  [2] R. Judson, “ToxValDB: Compiling Publicly Available In Vivo Toxicity Data,” 2019, The United States Environmental Protection Agency’s Center for Computational Toxicology and Exposure. doi: 10.23645/EPACOMPTOX.7800653  [3] S. Creton et al., “Acute toxicity testing of chemicals—Opportunities to avoid redundant testing and use alternative approaches,” Crit. Rev. Toxicol., vol. 40, no. 1, pp. 50–83, Jan. 2010, doi: 10.3109/10408440903401511 |
| 3.7. | Endpoint data quality and variability | Complete dataset:   \| Toxics \| Non-toxics \| Toxics / non- toxics \| \| --- \| --- \| --- \| \| 382 \| 2234 \| 0.17 \|   Balanced dataset:   \| Toxics \| Non- toxics \| Toxics / non- toxics \| \| --- \| --- \| --- \| \| 382 \| 382 \| 1.0 \| |
| **4** | **Defining the algorithm - OECD Principle 2 : “AN UNAMBIGUOUS ALGORITHM”** | **PRINCIPLE 2: “AN UNAMBIGUOUS ALGORITHM”. The (Q)SAR estimate of an endpoint is the result of applying an ALGORITHM to a set of structural parameters which describe the chemical structure. The intent of PRINCIPLE 2 (a (Q)SAR should be associated with an unambiguous algorithm) is to ensure transparency in the model algorithm that generates predictions of an endpoint from information on chemical structure and/or physicochemical properties. In this context, algorithm refers to any mathematical equation, decision rule or output approach.** |
| 4.1. | Type of model | Fully connected neural network |
| 4.2. | Explicit algorithm | Learning rate: 1e-3  Batch size: 32  Number of nodes of first dense layer: 256  Number of nodes of second dense layer: 128  Dropout rate for the first dropout layer after the first dense layer: 0.2  Dropout rate for the second dropout layer after the second dense layer: 0.1 |
| 4.3. | Descriptors in the model | The compounds were represented as Morgan fingerprints, MACCS keys, and Mordred descriptors. |
| 4.4. | Descriptor selection | The Morgan fingerprints and MACCS keys were used as bit vectors to represent the model.  A combination of univariate and multivariate selection methods was then applied to pinpoint the most informative Mordred descriptors. First, an Analysis of Variance (ANOVA) was performed to identify descriptors with statistically significant differences between classes (e.g., toxic vs. non-toxic). Subsequently, L1-penalized linear regression (also known as Lasso regression) was used to further reduce descriptor complexity by imposing sparsity, effectively zeroing out less relevant coefficients and producing a concise set of Mordred descriptors. |
| 4.5. | Algorithm and descriptor generation | RDKit: We utilized RDKit Open-Source Toolkit for Cheminformatics for MACCS keys and Morgan Fingerprints generation [1]  [1] RDKit, RDKit: Open-source cheminformatics. Accessed: May 02, 2025. [Online]. Available: <https://www.rdkit.org/>  Mordred: We utilized Mordred package for molecular descriptor calculation. Mordred descriptors [2] constitute a set of more than 1,800 two- and three-dimensional descriptors that capture geometric, electronic, topological, and hybrid characteristics. In this study, only the two-dimensional Mordred descriptors were utilized to ensure consistency with other molecular representations.  [2] H. Moriwaki, Y.-S. Tian, N. Kawashita, and T. Takagi, “Mordred: a molecular descriptor calculator,” J. Cheminformatics, vol. 10, no. 1, p. 4, Dec. 2018, doi: 10.1186/s13321-018-0258-y |
| 4.6. | Software name and version for descriptor generation | RDKit v. 2024.09.6  Mordred v. 2.0.6 |
| 4.7. | Chemicals/Descriptors ratio | Our dataset contains 764 chemicals. The model uses a combined input feature space of 2278 descriptors, comprising of 2048-bit Morgan fingerprint vectors, 167-bit MACCS keys vectors and 69 Mordred descriptors. While the ratio of chemicals/descriptors should be high in classical QSPR modeling (due to risk of overfitting when the number of descriptors exceeds the number of chemicals), this is not a limiting factor in deep learning approaches. Fully Connected Neural Networks handle high-dimensional input spaces by learning complex representations. The number of hidden neurons in the second layer 128 which then pass through the output layer for the final classification of the compounds. |
| **5** | **Defining the applicability domain - OECD Principle 3: “A DEFINED DOMAIN OF APPLICABILITY”** | **PRINCIPLE 3: “A DEFINED DOMAIN OF APPLICABILITY”. APPLICABILITY DOMAIN refers to the response and chemical structure space in which the model makes predictions with a given reliability. Ideally the applicability domain should express the structural, physicochemical and response space of the model. The CHEMICAL STRUCTURE (x variable) space can be expressed by information on physicochemical properties and/or structural fragments. The RESPONSE (y variable) can be any physicochemical, biological or environmental effect that is being predicted. According to PRINCIPLE 3 a (Q)SAR should be associated with a defined domain of applicability. Section 5 can be repeated (e.g., 5.a, 5.b, 5.c, etc) as many times as necessary if more than one method has been used to assess the applicability domain.** |
| 5.1. | Description of the applicability domain of the model | In this study, two approaches were used: a Euclidean distance-based method for Mordred descriptor features and a Tanimoto similarity-based method for MACCS keys and Morgan fingerprints.  For Euclidean distance-based method, the Euclidean distance between the test compound and the centroid in training data is calculated and if this value is larger than the calculated APD threshold, the test compound is considered outside the applicability domain.  For similarity-based method, if the average Tanimoto similarity between the test compound and its k most similar compounds in the training exceed the APD threshold calculated on the training compounds, then is considered to be in the applicability domain.  For the models trained on concatenated features -combining Mordred descriptors and fingerprints- we considered both approaches independently to evaluate their individual contribution to the AD. Specifically, we applied the Euclidean distance-based approach to the descriptor subset and the similarity-based approach to the fingerprint subset, both MACCS and Morgan fingerprints. To determine whether a compound falls within the AD of the model, we adopted a consensus approach, considering a compound inside the AD if it meets at least two of the three defined thresholds. |
| 5.2. | Method used to assess the applicability domain | For the Euclidean distance-based AD, the centroid of the training compounds was calculated as the mean of each scaled descriptor. The distances between all training compounds and the centroid were calculated, and the largest distance was adopted as the threshold $d_{t}$. Then, the Euclidean distance $d_{Euc,i}$ between the test compound $i$ and the centroid was calculated according to:  $d_{Euc,i}=\sqrt{\sum\left( x_{j,i}-x_{j, centroid} \right)^{2}}$  where $x_{j,i}$is the $j^{th}$ scaled descriptor of compound $i$ and $x_{j,centroid}$ is the $j^{th}$ scaled descriptor of the centroid. In case the $d_{Euc,i}$ of compound $i$ is larger than the $d_{t}$, the compound is considered outside the AD.  The similarity-based AD was adopted for the fingerprint representation of compounds. This methodology computes the similarity matrix between the training compounds and the test compounds. A similarity threshold is calculated as defined:  $S_{T}=\bar{\gamma}+Z\sigma$  where $\bar{\gamma}$ represents the average Tanimoto similarity between training compounds, $\sigma$ is the standard deviation of the computed Tanimoto similarities, and $Z$ is a predefined value indicating the significance level. If the average Tanimoto similarity between the test compound and its $k$ most similar compounds in the training set exceeds the threshold $S_{T}$, it is consided to be in the AD, otherwise the compound is considered outside the AD. |
| 5.3. | Software name and version for applicability domain assessment | Python v. 3.10 |
| 5.4. | Limits of applicability | Euclidean-based APD threshold (Mordred descriptors): 37.219  Similarity-based APD threshold (MACCS keys): 0.354  Similarity-based APD threshold (Morgan Fingerprints): 0.137 |
| **6** | **Defining goodness-of-fit and robustness (internal validation) – OECD Principle 4: “APPROPRIATE MEASURES OF GOODNESS-OF-FIT, ROBUSTENESS AND PREDICTIVITY”** | **PRINCIPLE 4: “APPROPRIATE MEASURES OF GOODNESS-OF-FIT, ROBUSTENESS AND PREDICTIVITY”. PRINCIPLE 4 expresses the need to perform validation to establish the performance of the model. GOODNESS-OF-FIT and ROBUSTNESS refer to the internal model performance.** |
| 6.1. | Availability of the training set | Available at the ChemPharos database:  https://db.chempharos.eu/datasets/Datasets.zul |
| 6.2. | Available information for the training set | a) Chemical names (common names and/or IUPAC names); b) CAS numbers; c) SMILES; d) InChI codes; e) Structural formula; f) MW |
| 6.3. | Data for each descriptor variable for the training set | Available at the ChemPharos database:  https://db.chempharos.eu/datasets/Datasets.zul |
| 6.4. | Data for the dependent variable for the training set | Available at the ChemPharos database:  https://db.chempharos.eu/datasets/Datasets.zul |
| 6.5. | Other information about the training set | Random splitting (stratified based on the chemicals’ class) was used for partitioning: 611 out of 764 molecules were included in the training set for model development. From the remaining 153 molecules, 76 were randomly selected as the validation set for hyperparameter optimization and the other 77 molecules were used as a test set for evaluating model performance. |
| 6.6. | Pre-processing of data before modelling | Checking SMILES structures with RDKit molecular object.  Removal of columns containing NA values.  In case of unbalanced dataset, an undersampling strategy was employed to result in a balanced dataset.  Z-score normalization of descriptors.  Variable selection of Mordred descriptors was performed according to §4.4. |
| 6.7. | Statistics for goodness-of-fit | Training set:   \| **Metric** \| **Value** \| \| --- \| --- \| \| ACC \| 0.997 \| \| AUC \| 1.000 \| \| PRE \| 0.993 \| \| SPE \| 0.993 \| \| SEN \| 1.000 \| \| MCC \| 0.993 \| \| F1 \| 0.997 \| |
| 6.8. | Robustness - Statistics obtained by leave-one-out cross-validation | NA |
| 6.9. | Robustness - Statistics obtained by leave-many-out cross-validation | Robustness – Statistics obtained by 5-fold cross-validation (random splitting of data).   \| **Metric** \| **Value** \| \| --- \| --- \| \| ACC \| 0.849 \| \| AUC \| 0.923 \| \| PRE \| 0.847 \| \| SPE \| 0.846 \| \| SEN \| 0.851 \| \| MCC \| 0.698 \| \| F1 \| 0.849 \| |
| 6.10. | Robustness - Statistics obtained by Y-scrambling | \| **Iteration** \| **ACC** \| **AUC** \| **SEN** \| **SPE** \| **MCC** \| \| --- \| --- \| --- \| --- \| --- \| --- \| \| 1 \| 0.532 \| 0.551 \| 0.513 \| 0.553 \| 0.065 \| \| 2 \| 0.536 \| 0.523 \| 0.490 \| 0.584 \| 0.175 \| \| 3 \| 0.545 \| 0.561 \| 0.564 \| 0.526 \| 0.090 \| \| 4 \| 0.519 \| 0.536 \| 0.692 \| 0.342 \| 0.037 \| \| 5 \| 0.471 \| 0.518 \| 0.259 \| 0.689 \| 0.064 \| |
| 6.11. | Robustness - Statistics obtained by bootstrap | NA |
| 6.12. | Robustness - Statistics obtained by other methods | NA |
| **7** | **Defining predictivity (external validation) – OECD Principle 4: “APPROPRIATE MEASURES OF GOODNESS-OF-FIT, ROBUSTENESS AND PREDICTIVITY”** | **PRINCIPLE 4: “APPROPRIATE MEASURES OF GOODNESS-OF-FIT, ROBUSTENESS AND PREDICTIVITY”. PRINCIPLE 4 expresses the need to perform validation to establish the performance of the model. PREDICTIVITY refers to the external model validation. Section 7 can be repeated (e.g., 7.a, 7.b, 7.c, etc) as many times as necessary if more validation studies need to be reported in the QMRF.** |
| 7.1. | Availability of the external validation set | Available at the ChemPharos database:  https://db.chempharos.eu/datasets/Datasets.zul |
| 7.2. | Available information for the external validation set | a) Chemical names (common names and/or IUPAC names); b) CAS numbers; c) SMILES; d) InChI codes; e) Structural formula; f) MW |
| 7.3. | Data for each descriptor variable for the external validation set | Available at the ChemPharos database:  https://db.chempharos.eu/datasets/Datasets.zul |
| 7.4. | Data for the dependent variable for the external validation set | Available at the ChemPharos database:  https://db.chempharos.eu/datasets/Datasets.zul |
| 7.5. | Other information about the external validation set | 77 molecules were included in the external validation (test set), which was not involved in model development, but it was rather used solely for validating purposes. |
| 7.6. | Experimental design of test set | Randomly selected from the first partitioning of data (153 molecules). See also §6.5. |
| 7.7. | Predictivity - Statistics obtained by external validation | \| **Metric** \| **Value** \| \| --- \| --- \| \| ACC \| 0.792 \| \| AUC \| 0.823 \| \| PRE \| 0.848 \| \| SPE \| 0.868 \| \| SEN \| 0.718 \| \| MCC \| 0.592 \| \| F1 \| 0.778 \| |
| 7.8. | Predictivity - Assessment of the external validation set | The external validation set (test set) is 10% of the initial dataset, 100% of predictions fall within the domain of applicability. |
| 7.9. | Comments on the external validation of the model | Test and validation sets were normalized based on the Z-score normalization applied on the training set. |
| **8** | **Providing a mechanistic interpretation - OECD Principle 5: “A MECHANISTIC INTERPRETATION, IF POSSIBLE”** | **PRINCIPLE 5: “A MECHANISTIC INTERPRETATION, IF POSSIBLE”. According to PRINCIPLE 5, a (Q)SAR should be associated with a mechanistic interpretation, if possible.** |
| 8.1. | Mechanistic basis of the model | NA |
| 8.2. | A priori or a posteriori mechanistic interpretation | NA |
| 8.3. | Other information about the mechanistic interpretation | NA |
| **9** | **Miscellaneous information** |  |
| 9.1. | Comments | The dataset was from Borba *et al*. [1], of 764 chemicals from different sources.  [1] J. V. B. Borba et al., “STopTox: An in Silico Alternative to Animal Testing for Acute Systemic and Topical Toxicity,” Environ. Health Perspect., vol. 130, no. 2, p. 027012, Feb. 2022, doi: 10.1289/EHP9341 |
| 9.2. | Bibliography | 1. J. V. B. Borba et al., “STopTox: An in Silico Alternative to Animal Testing for Acute Systemic and Topical Toxicity,” Environ. Health Perspect., vol. 130, no. 2, p. 027012, Feb. 2022, doi: 10.1289/EHP9341 2. ECHA (European Chemical Agency), OECD (Organization for Economic Co- and operation and Development)., REACH Study Results - IUCLID, 2019. Accessed: Feb. 02, 2025. [Online]. Available: https://iuclid6.echa.europa.eu/reach-study-results |
| 9.3 | Supporting information | NA |

# **Section 7:** MODA for skin sensitization model

Moda powered by [**Enalos Cloud Platform**](http://www.enaloscloud.novamechanics.com/)

MODA for

Simulated in project

**Skin sensitization prediction**

| **OVERVIEW of the SIMULATION** | | | |
| --- | --- | --- | --- |
| **1** | **USER CASE** | Skin sensitization prediction | |
| **2** | **CHAIN OF MODELS** | **Data Transformation 1** | Fingerprint-based model Data Transformation |
|  |  | **Data Transformation 2** | Fingerprint-based model Data Transformation |
|  |  | **Data Transformation 3** | Descriptor-based model Data Transformation |
|  |  | **Model 4** | Prediction model Data based Model |
|  |  | **Model 5** | Applicability Domain model Data based Model |
| **3** | **PUBLICATION PEER - REVIEWING THE DATA** | DOI provided: No | |
| **4** | **ACCESS CONDITIONS** | Access type: Free Owner of workflow: Workflow access link: | |
| **5** | **WORKFLOW AND ITS RATIONALE** |  | |

**Workflow picture**


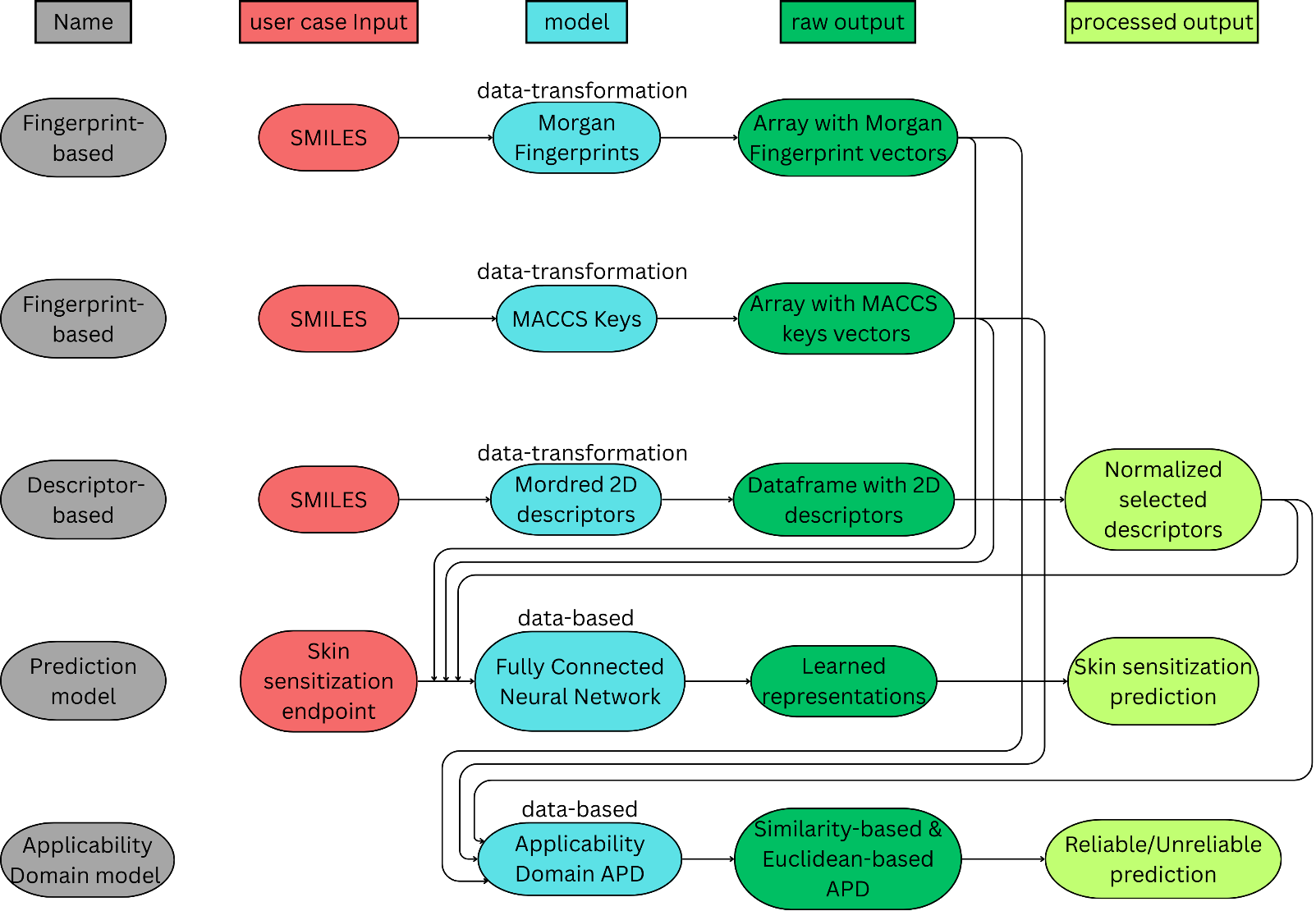


**Each physics-based model used in this simulation is to be documented in four chapters:**

1. Aspect of the User Case or system simulated with this model
2. Model: Please make sure the notions Physics Equation and Materials Relation are properly understood.
   - Tightly coupled models can be written up collectively in one set of four tables. To solve tightly coupled PE one matrix is set up and solved in one go.
   - For continuum models the PE is often the conservation equations coded up in bought software packages.
   - Often the MR is established by the modeller.
3. Computational aspects include also a documentation of how the user case specifications are translated into computer language.
4. Post processing documents how the raw output of one simulation is processed into input for the next simulation. This information given under 4.1 in the first model will be the same as the "simulated input" information under 2.4 for the next model. This is the essence of model inter- operability!
5. Pre-processing before the first model can be depicted in pink as it is considered to be part of the user-case.

**Each data-based model in this simulation is to be documented in three chapters:**

1. Aspect of the User Case or system simulated with this data-based model
2. Data-based Model
3. Computational detail of the datamining operation

**Data Transformation 1**

Fingerprint-based model

| **Aspect of the User Case/System** | | |
| --- | --- | --- |
| **1.1** | **Aspect of the User Case to be simulated** | Convert SMILES to Morgan Fingerprints |
| **1.2** | **Material** | Small organic molecules |
| **1.3** | **Geometry** |  |
| **1.4** | **Time Lapse** |  |
| **1.5** | **Manufacturing process or in- service conditions** |  |
| **1.6** | **Publication on this data** | Pending publication |

| **Data Transformation** | | | |
| --- | --- | --- | --- |
| **2.0** | **Equation type and name** |  | |
| **2.1** | **Database and type** |  | |
| **2.2** | **Equation** | Hypothesis |  |
|  |  | Physical quantities |  |

| **Computational detail** | | |
| --- | --- | --- |
| **3.1** | **Numerical Operations** |  |
| **3.2** | **Software tool** | RDKit v. 2024.09.6 |
| **3.3** | **Margin Of Error** |  |

**Data Transformation 2**

Fingerprint-based model

| **Aspect of the User Case/System** | | |
| --- | --- | --- |
| **1.1** | **Aspect of the User Case to be simulated** | Convert SMILES to MACCS Keys |
| **1.2** | **Material** | Small organic molecules |
| **1.3** | **Geometry** |  |
| **1.4** | **Time Lapse** |  |
| **1.5** | **Manufacturing process or in- service conditions** |  |
| **1.6** | **Publication on this data** | Pending publication |

| **Data Transformation** | | | |
| --- | --- | --- | --- |
| **2.0** | **Equation type and name** |  | |
| **2.1** | **Database and type** |  | |
| **2.2** | **Equation** | Hypothesis |  |
|  |  | Physical quantities |  |

| **Computational detail** | | |
| --- | --- | --- |
| **3.1** | **Numerical Operations** |  |
| **3.2** | **Software tool** | RDKit v. 2024.09.6 |
| **3.3** | **Margin Of Error** |  |

**Data Transformation 3**

Descriptor-based model

| **Aspect of the User Case/System** | | |
| --- | --- | --- |
| **1.1** | **Aspect of the User Case to be simulated** | Convert SMILES to Mordred Descriptors |
| **1.2** | **Material** | Small organic molecules |
| **1.3** | **Geometry** |  |
| **1.4** | **Time Lapse** |  |
| **1.5** | **Manufacturing process or in- service conditions** |  |
| **1.6** | **Publication on this data** | Pending publication |

| **Data Transformation** | | | |
| --- | --- | --- | --- |
| **2.0** | **Equation type and name** |  | |
| **2.1** | **Database and type** |  | |
| **2.2** | **Equation** | Hypothesis |  |
|  |  | Physical quantities |  |

| **Computational detail** | | |
| --- | --- | --- |
| **3.1** | **Numerical Operations** |  |
| **3.2** | **Software tool** | Mordred v. 2.0.6 |
| **3.3** | **Margin Of Error** |  |

**Model 4**

Prediction model

| **Aspect of the User Case/System to be Simulated** | | |
| --- | --- | --- |
| **1.1** | **Aspect of the User Case to be simulated** | Skin sensitization chemical records with LLNA data  Skin sensitization is the most common immune response experienced in humans. It occures when a person is exposed to sensitizing chemicals, which may lead to allergic contact fermatitis. Evaluating the skin sensitization potential is essential for safety assessment of chemicals.  Endpoint Units: NA  Skin sensitization data were collected from the National Toxicology Program Interagency Center for the Evaluation of Alternative Toxicological Methods on behalf of ICCVAM [1] and the Registration,  Evaluation, Authorization and restriction of Chemicals (REACH) Study Results Database [2].   1. ICCVAM (Interagency Coordinating Committee on the Validation of Alternative and Methods), NICEATM Murine Local Lymph Node Assay (LLNA) Database, 2013. Accessed: Feb. 02, 2025. [Online]. Available: https://ntp.niehs.nih.gov/whatwestudy/niceatm/test-method- evaluations/skin- sens/llna/index.html 2. ECHA (European Chemical Agency), OECD (Organization for Economic Co- and operation and Development)., REACH Study Results   - IUCLID, 2019. Accessed: Feb. 02, 2025. [Online]. Available: https://iuclid6.echa.europa.eu/reach-study-results  Complete dataset: Sensitizers: 481  Non-sensitizers: 519  Sensitizers / non-sensitizers: 0.93 |

| **1.2** | **Material** | Small organic molecules |
| --- | --- | --- |
| **1.3** | **Geometry** |  |
| **1.4** | **Time Lapse** |  |
| **1.5** | **Manufacturing process or in- service conditions** |  |
| **1.6** | **Publication on this data** | Pending publication |

| **The Data-based Model** | | |
| --- | --- | --- |
| **2.0** | **Equation type and name** | Explicit algorithm:  Learning rate: 1e-3, Batch size: 32, Number of nodes of first dense  layer: 1024, Number of nodes of second dense layer: 256, Dropout rate for the first dropout layer after the first dense layer: 0.3, Dropout rate for the second dropout layer after the second dense layer: 0.1  Availability of the datasets:  Available at the ChemPharos database: <https://db.chempharos.eu/datasets/Datasets.zul>  Available information for the datasets:   1. Chemical names (common names and/or IUPAC names) 2. CAS numbers 3. SMILES 4. InChI codes 5. Structural formula 6. MW   Pre-processing of data before modelling:  Checking SMILES structures with RDKit molecular object. Removal of columns containing NA values. In case of unbalanced dataset, an undersampling strategy was employed to result in a balanced dataset. Z-score normalization of descriptors. Variable selection of Mordred descriptors.  Availability of the external validation set:  Available at the ChemPharos database: <https://db.chempharos.eu/datasets/Datasets.zul>  Available information for the external validation set:   1. Chemical names (common names and/or IUPAC names) 2. CAS numbers 3. SMILES 4. InChI codes 5. Structural formula 6. MW   Other information about the external validation set:  100 molecules were included in the external validation (test set), which was not involved in model development, but it was rather used solely for validating purposes.  Experimental design of test set:  Randomly selected from the first partitioning of data (200 molecules). |

| **2.1** | **Database and type** |  | |
| --- | --- | --- | --- |
| **2.2** | **Equation** | Hypothesis | Fully connected neural network |
|  |  | Physical quantities | Descriptors in the model: The compounds were represented as Morgan fingerprints, MACCS keys, and Mordred descriptors.  Descriptor selection: The Morgan fingerprints and MACCS keys were used as bit vectors to represent the model. A combination of univariate and multivariate selection methods was then applied to pinpoint the most informative Mordred descriptors. First, an Analysis of Variance (ANOVA) was performed to identify descriptors with statistically significant differences between classes (e.g., sensitizers vs. non-sensitizers). Subsequently, L1-penalized linear regression (also known as Lasso regression) was used to further reduce descriptor complexity by imposing sparsity, effectively zeroing out less relevant coefficients and producing a concise set of Mordred descriptors.  Algorithm and descriptor generation: RDKit: We utilized RDKit Open-Source Toolkit for Cheminformatics for MACCS keys and Morgan Fingerprints generation [1]  [1] RDKit, RDKit: Open-source cheminformatics. Accessed: May 02, 2025. [Online]. Available: https://www.rdkit.org/  Mordred: We utilized Mordred package for molecular descriptor calculation. Mordred descriptors [2] constitute a set of more than 1,800 two- and three-dimensional descriptors that capture geometric, electronic, topological, and hybrid characteristics. In this study, only the two-dimensional Mordred descriptors were utilized to ensure consistency with other molecular representations.  [2] H. Moriwaki, Y.-S. Tian, N. Kawashita, and T. Takagi, “Mordred: a molecular descriptor calculator,” J. Cheminformatics, vol. 10, no. 1, p. 4, Dec. 2018, doi: 10.1186/s13321-018-0258-y  Software name and version for descriptor and algorithm generation:  RDKit v. 2024.09.6  Mordred v. 2.0.6  Chemicals/ Descriptors ratio: Our dataset contains 1000 chemicals. The model uses a combined input feature space of 2278 descriptors, comprising of 2048-bit Morgan fingerprint vectors, 167-bit MACCS keys vectors and 63 Mordred descriptors. While the ratio of chemicals/descriptors should be high in classical QSPR modeling (due to risk of overfitting when the number of descriptors exceeds the number of chemicals), this is not a limiting factor in deep learning approaches. Fully Connected Neural Networks handle high-dimensional input spaces by learning complex representations. The number of hidden neurons in the second layer 256 which then pass through the output layer for the final classification of the compounds. |

| **Computational detail of datamining operation** | | |
| --- | --- | --- |
| **3.1** | **Numerical Operations** | Explicit Algorithm:  Learning rate: 1e-3 Batch size: 32  Number of nodes of first dense layer: 1024 Number of nodes of second dense layer: 256  Dropout rate for the first dropout layer after the first dense layer: 0.3 Dropout rate for the second dropout layer after the second dense  layer: 0.1 |
| **3.2** | **Software tool** | Python v. 3.10 |
| **3.3** | **Margin Of Error** | Statistics for goodness-of-fit:  ACC: 0.981  AUC: 0.997  PRE: 0.967  SPE: 0.969  SEN: 0.995  MCC: 0.963  F1: 0.981  Robustness – Statistics obtained by 5-fold cross-validation (random  splitting of data):  ACC: 0.721  AUC: 0.792  PRE: 0.699  SPE: 0.704  SEN: 0.739  MCC: 0.444  F1: 0.718  Y-randomization:   \| Iteration \| ACC \| AUC \| SEN \| SPE \| MCC \| \| --- \| --- \| --- \| --- \| --- \| --- \| \| 1 \| 0.540 \| 0.527 \| 0.667 \| 0.423 \| 0.092 \| \| 2 \| 0.530 \| 0.515 \| 0.438 \| 0.615 \| 0.054 \| \| 3 \| 0.440 \| 0.445 \| 0.583 \| 0.308 \| -0.113 \| \| 4 \| 0.560 \| 0.595 \| 0.604 \| 0.519 \| 0.124 \| \| 5 \| 0.440 \| 0.465 \| 0.375 \| 0.500 \| -0.126 \|   Predictivity - Statistics obtained by external validation:  ACC: 0.85  AUC: 0.881  PRE: 0.851  SPE: 0.865  SEN: 0.833  MCC: 0.699  F1: 0.842 |

**Model 5**

Applicability Domain model

| **Aspect of the User Case/System to be Simulated** | | |
| --- | --- | --- |
| **1.1** | **Aspect of the User Case to be simulated** |  |
| **1.2** | **Material** |  |
| **1.3** | **Geometry** |  |
| **1.4** | **Time Lapse** |  |
| **1.5** | **Manufacturing process or in- service conditions** | In this study, two approaches were used: a Euclidean distance-based method for Mordred descriptor features and a Tanimoto similarity-based method for MACCS keys and Morgan fingerprints.  For Euclidean distance-based method, the Euclidean distance between the test compound and the centroid in training data is calculated and if this value is larger than the calculated APD threshold, the test compound is considered outside the applicability domain.  For similarity-based method, if the average Tanimoto similarity between the test compound and its k most similar compounds in the training exceed the APD threshold calculated on the training compounds, then is considered to be in the applicability domain.  For the models trained on concatenated features -combining Mordred descriptors and fingerprints- we considered both approaches independently to evaluate their individual contribution to the AD. Specifically, we applied the Euclidean distance-based approach to the descriptor subset and the similarity-based approach to the fingerprint subset, both MACCS and Morgan fingerprints. To determine whether a compound falls within the AD of the model, we adopted a consensus approach, considering a compound inside the AD if it meets at least two of the three defined thresholds.  For the Euclidean distance-based AD, the centroid of the training compounds was calculated as the mean of each scaled descriptor. The distances between all training compounds and the centroid were calculated, and the largest distance was adopted as the threshold d_t_.  Then, the Euclidean distance d_Euc,i_ between the test compound i and |

| **Aspect of the User Case/System to be Simulated** | | |
| --- | --- | --- |
|  |  | the centroid was calculated.  In case the d_Euc,i_ of compound i is larger than the d_t_, the compound is considered outside the AD.  The similarity-based AD was adopted for the fingerprint representation of compounds. This methodology computes the similarity matrix between the training compounds and the test compounds. If the average Tanimoto similarity between the test compound and its k most similar compounds in the training set exceeds the threshold S_T_, it is considered to be in the AD, otherwise the compound is considered outside the AD.  Software name for applicability domain assessment: Python v. 3.10 Limits of applicability domain:  Euclidean-based APD threshold (Mordred descriptors): 38.193 Similarity-based APD threshold (MACCS keys): 0.332  Similarity-based APD threshold (Morgan Fingerprints): 0.148 |
| **1.6** | **Publication on this data** | Pending publication |

| **The Data-based Model** | | | |
| --- | --- | --- | --- |
| **2.0** | **Equation type and name** |  | |
| **2.1** | **Database and type** |  | |
| **2.2** | **Equation** | Hypothesis |  |
|  |  | Physical quantities |  |

| **Computational detail of datamining operation** | | |
| --- | --- | --- |
| **3.1** | **Numerical Operations** |  |
| **3.2** | **Software tool** | Python v. 3.10 |
| **3.3** | **Margin Of Error** |  |

# **Section 8:** MODA for skin irritation/corrosion model

Moda powered by [**Enalos Cloud Platform**](http://www.enaloscloud.novamechanics.com/)

MODA for

Simulated in project

**Skin irritation/corrosion prediction**

| **OVERVIEW of the SIMULATION** | | | |
| --- | --- | --- | --- |
| **1** | **USER CASE** | Skin irritation/corrosion prediction | |
| **2** | **CHAIN OF MODELS** | **Data Transformation 1** | Fingerprint-based model Data Transformation |
|  |  | **Data Transformation 2** | Fingerprint-based model Data Transformation |
|  |  | **Data Transformation 3** | Descriptor-based model Data Transformation |
|  |  | **Model 4** | Prediction model Data based Model |
|  |  | **Model 5** | Applicability Domain model Data based Model |
| **3** | **PUBLICATION PEER - REVIEWING THE DATA** | DOI provided: No | |
| **4** | **ACCESS CONDITIONS** | Access type: Free Owner of workflow: Workflow access link: | |
| **5** | **WORKFLOW AND ITS RATIONALE** |  | |

**Workflow picture**


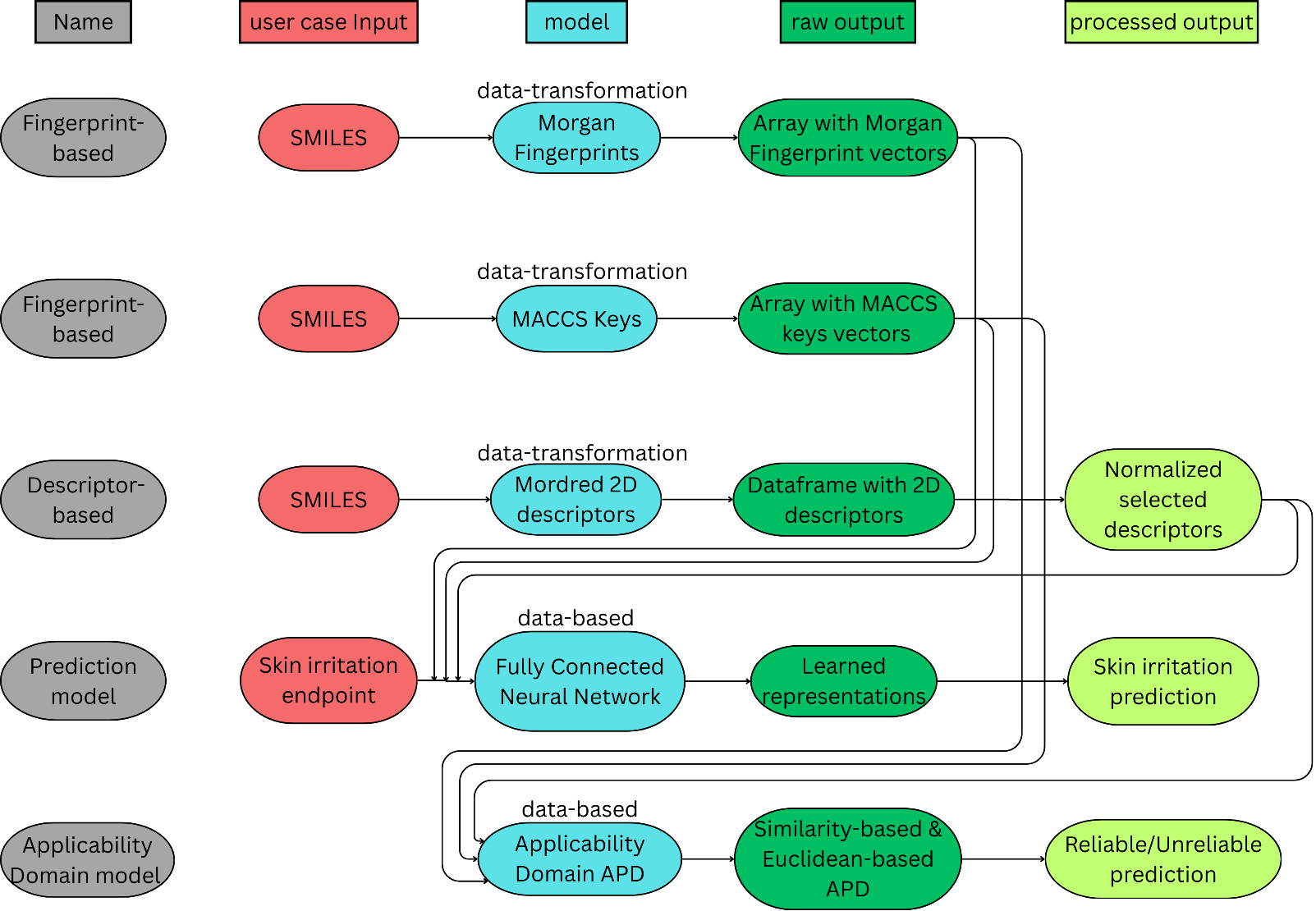


**Each physics-based model used in this simulation is to be documented in four chapters:**

1. Aspect of the User Case or system simulated with this model
2. Model: Please make sure the notions Physics Equation and Materials Relation are properly understood.
   1. Tightly coupled models can be written up collectively in one set of four tables. To solve tightly coupled PE one matrix is set up and solved in one go.
   2. For continuum models the PE is often the conservation equations coded up in bought software packages.
   3. Often the MR is established by the modeller.
3. Computational aspects include also a documentation of how the user case specifications are translated into computer language.
4. Post processing documents how the raw output of one simulation is processed into input for the next simulation. This information given under 4.1 in the first model will be the same as the "simulated input" information under 2.4 for the next model. This is the essence of model inter- operability!
5. Pre-processing before the first model can be depicted in pink as it is considered to be part of the user-case.

**Each data-based model in this simulation is to be documented in three chapters:**

1. Aspect of the User Case or system simulated with this data-based model
2. Data-based Model
3. Computational detail of the datamining operation

**Data Transformation 1**

Fingerprint-based model

| **Aspect of the User Case/System** | | |
| --- | --- | --- |
| **1.1** | **Aspect of the User Case to be simulated** | Convert SMILES to Morgan Fingerprints |
| **1.2** | **Material** | Small organic molecules |
| **1.3** | **Geometry** |  |
| **1.4** | **Time Lapse** |  |
| **1.5** | **Manufacturing process or in- service conditions** |  |
| **1.6** | **Publication on this data** | Pending publication |

| **Data Transformation** | | | |
| --- | --- | --- | --- |
| **2.0** | **Equation type and name** |  | |
| **2.1** | **Database and type** |  | |
| **2.2** | **Equation** | Hypothesis |  |
|  |  | Physical quantities |  |

| **Computational detail** | | |
| --- | --- | --- |
| **3.1** | **Numerical Operations** |  |
| **3.2** | **Software tool** | RDKit v. 2024.09.6 |
| **3.3** | **Margin Of Error** |  |

**Data Transformation 2**

Fingerprint-based model

| **Aspect of the User Case/System** | | |
| --- | --- | --- |
| **1.1** | **Aspect of the User Case to be simulated** | Convert SMILES to MACCS Keys |
| **1.2** | **Material** | Small organic molecules |
| **1.3** | **Geometry** |  |
| **1.4** | **Time Lapse** |  |
| **1.5** | **Manufacturing process or in- service conditions** |  |
| **1.6** | **Publication on this data** | Pending publication |

| **Data Transformation** | | | |
| --- | --- | --- | --- |
| **2.0** | **Equation type and name** |  | |
| **2.1** | **Database and type** |  | |
| **2.2** | **Equation** | Hypothesis |  |
|  |  | Physical quantities |  |

| **Computational detail** | | |
| --- | --- | --- |
| **3.1** | **Numerical Operations** |  |
| **3.2** | **Software tool** | RDKit v. 2024.09.6 |
| **3.3** | **Margin Of Error** |  |

**Data Transformation 3**

Descriptor-based model

| **Aspect of the User Case/System** | | |
| --- | --- | --- |
| **1.1** | **Aspect of the User Case to be simulated** | Convert SMILES to Mordred Descriptors |
| **1.2** | **Material** | Small organic molecules |
| **1.3** | **Geometry** |  |
| **1.4** | **Time Lapse** |  |
| **1.5** | **Manufacturing process or in- service conditions** |  |
| **1.6** | **Publication on this data** | Pending publication |

| **Data Transformation** | | | |
| --- | --- | --- | --- |
| **2.0** | **Equation type and name** |  | |
| **2.1** | **Database and type** |  | |
| **2.2** | **Equation** | Hypothesis |  |
|  |  | Physical quantities |  |

| **Computational detail** | | |
| --- | --- | --- |
| **3.1** | **Numerical Operations** |  |
| **3.2** | **Software tool** | Mordred v. 2.0.6 |
| **3.3** | **Margin Of Error** |  |

**Model 4**

Prediction model

| **Aspect of the User Case/System to be Simulated** | | |
| --- | --- | --- |
| **1.1** | **Aspect of the User Case to be simulated** | Skin irritation/corrosion chemical records  Skin irritation is a significant adverse effect associated with chemicals and drug substances, which poses significant challenges in dermatology.  Endpoint Units: NA  Skin irritation/corrosion data were collected from the Registration, Evaluation, Authorization and restriction of Chemicals (REACH) Study Results Database [2].  [2] ECHA (European Chemical Agency), OECD (Organization for Economic Co- and operation and Development)., REACH Study Results - IUCLID, 2019. Accessed: Feb. 02, 2025. [Online]. Available: <https://iuclid6.echa.europa.eu/reach-study-results>  Complete dataset:  Irritants: 317  Non-irritants: 694  Irritants / non- irritants: 0.46  Balanced dataset:  Irritants: 317  Non- irritants: 317  Irritants / non- irritants: 1.0 |

| **1.2** | **Material** | Small organic molecules |
| --- | --- | --- |
| **1.3** | **Geometry** |  |
| **1.4** | **Time Lapse** |  |
| **1.5** | **Manufacturing process or in- service conditions** |  |
| **1.6** | **Publication on this data** | Pending publication |

| **The Data-based Model** | | |
| --- | --- | --- |
| **2.0** | **Equation type and name** | Explicit algorithm:  Learning rate: 1e-4, Batch size: 32, Number of nodes of first dense  layer: 512, Number of nodes of second dense layer: 256, Dropout rate for the first dropout layer after the first dense layer: 0.3, Dropout rate for the second dropout layer after the second dense layer: 0.1  Availability of the datasets:  Available at the ChemPharos database: <https://db.chempharos.eu/datasets/Datasets.zul>  Available information for the datasets:   1. Chemical names (common names and/or IUPAC names) 2. CAS numbers 3. SMILES 4. InChI codes 5. Structural formula 6. MW   Pre-processing of data before modelling:  Checking SMILES structures with RDKit molecular object. Removal of columns containing NA values. In case of unbalanced dataset, an undersampling strategy was employed to result in a balanced dataset. Z-score normalization of descriptors. Variable selection of Mordred descriptors.  Availability of the external validation set:  Available at the ChemPharos database: <https://db.chempharos.eu/datasets/Datasets.zul>  Available information for the external validation set:   1. Chemical names (common names and/or IUPAC names) 2. CAS numbers 3. SMILES 4. InChI codes 5. Structural formula 6. MW   Other information about the external validation set:  64 molecules were included in the external validation (test set), which was not involved in model development, but it was rather used solely for validating purposes.  Experimental design of test set:  Randomly selected from the first partitioning of data (127 molecules). |

| **2.1** | **Database and type** |  | |
| --- | --- | --- | --- |
| **2.2** | **Equation** | Hypothesis | Fully connected neural network |
|  |  | Physical quantities | Descriptors in the model: The compounds were represented as Morgan fingerprints, MACCS keys, and Mordred descriptors.  Descriptor selection: The Morgan fingerprints and MACCS keys were used as bit vectors to represent the model. A combination of univariate and multivariate selection methods was then applied to pinpoint the most informative Mordred descriptors. First, an Analysis of Variance (ANOVA) was performed to identify descriptors with statistically significant differences between classes (e.g., irritants vs. non- irritants). Subsequently, L1-penalized linear regression (also known as Lasso regression) was used to further reduce descriptor complexity by imposing sparsity, effectively zeroing out less relevant coefficients and producing a concise set of Mordred descriptors.  Algorithm and descriptor generation: RDKit: We utilized RDKit Open-Source Toolkit for Cheminformatics for MACCS keys and Morgan Fingerprints generation [1]  [1] RDKit, RDKit: Open-source cheminformatics. Accessed: May 02, 2025. [Online]. Available: https://www.rdkit.org/  Mordred: We utilized Mordred package for molecular descriptor calculation. Mordred descriptors [2] constitute a set of more than 1,800 two- and three-dimensional descriptors that capture geometric, electronic, topological, and hybrid characteristics. In this study, only the two-dimensional Mordred descriptors were utilized to ensure consistency with other molecular representations.  [2] H. Moriwaki, Y.-S. Tian, N. Kawashita, and T. Takagi, “Mordred: a molecular descriptor calculator,” J. Cheminformatics, vol. 10, no. 1, p. 4, Dec. 2018, doi: 10.1186/s13321-018-0258-y  Software name and version for descriptor and algorithm generation:  RDKit v. 2024.09.6  Mordred v. 2.0.6  Chemicals/ Descriptors ratio: Our dataset contains 634 chemicals. The model uses a combined input feature space of 2278 descriptors, comprising of 2048-bit Morgan fingerprint vectors, 167-bit MACCS keys vectors and 62 Mordred descriptors. While the ratio of chemicals/descriptors should be high in classical QSPR modeling (due to risk of overfitting when the number of descriptors exceeds the number of chemicals), this is not a limiting factor in deep learning approaches. Fully Connected Neural Networks handle high-dimensional input spaces by learning complex representations. The number of hidden neurons in the second layer 256 which then pass through the output layer for the final classification of the compounds. |

| **Computational detail of datamining operation** | | |
| --- | --- | --- |
| **3.1** | **Numerical Operations** | Explicit Algorithm:  Learning rate: 1e-4 Batch size: 32  Number of nodes of first dense layer: 512  Number of nodes of second dense layer: 256  Dropout rate for the first dropout layer after the first dense layer: 0.3 Dropout rate for the second dropout layer after the second dense  layer: 0.1 |
| **3.2** | **Software tool** | Python v. 3.10 |
| **3.3** | **Margin Of Error** | Statistics for goodness-of-fit:  ACC: 0.947  AUC: 0.991  PRE: 0.909  SPE: 0.902  SEN: 0.992  MCC: 0.897  F1: 0.949  Robustness – Statistics obtained by 5-fold cross-validation (random  splitting of data):  ACC: 0.817  AUC: 0.883  PRE: 0.784  SPE: 0.754  SEN: 0.754  MCC: 0.643  F1: 0.828  Y-randomization:   \| Iteration \| ACC \| AUC \| SEN \| SPE \| MCC \| \| --- \| --- \| --- \| --- \| --- \| --- \| \| 1 \| 0.509 \| 0.514 \| 0.619 \| 0.400 \| 0.124 \| \| 2 \| 0.484 \| 0.510 \| 0.281 \| 0.688 \| -0.034 \| \| 3 \| 0.516 \| 0.491 \| 0.250 \| 0.781 \| 0.037 \| \| 4 \| 0.500 \| 0.494 \| 0.313 \| 0.688 \| 0.000 \| \| 5 \| 0.469 \| 0.426 \| 0.469 \| 0.469 \| -0.063 \|   Predictivity - Statistics obtained by external validation:  ACC: 0.859  AUC: 0.905  PRE: 0.871  SPE: 0.875  SEN: 0.844  MCC: 0.719  F1: 0.857 |

**Model 5**

Applicability Domain model

| **Aspect of the User Case/System to be Simulated** | | |
| --- | --- | --- |
| **1.1** | **Aspect of the User Case to be simulated** |  |
| **1.2** | **Material** |  |
| **1.3** | **Geometry** |  |
| **1.4** | **Time Lapse** |  |
| **1.5** | **Manufacturing process or in- service conditions** | In this study, two approaches were used: a Euclidean distance-based method for Mordred descriptor features and a Tanimoto similarity-based method for MACCS keys and Morgan fingerprints.  For Euclidean distance-based method, the Euclidean distance between the test compound and the centroid in training data is calculated and if this value is larger than the calculated APD threshold, the test compound is considered outside the applicability domain.  For similarity-based method, if the average Tanimoto similarity between the test compound and its k most similar compounds in the training exceed the APD threshold calculated on the training compounds, then is considered to be in the applicability domain.  For the models trained on concatenated features -combining Mordred descriptors and fingerprints- we considered both approaches independently to evaluate their individual contribution to the AD. Specifically, we applied the Euclidean distance-based approach to the descriptor subset and the similarity-based approach to the fingerprint subset, both MACCS and Morgan fingerprints. To determine whether a compound falls within the AD of the model, we adopted a consensus approach, considering a compound inside the AD if it meets at least two of the three defined thresholds.  For the Euclidean distance-based AD, the centroid of the training compounds was calculated as the mean of each scaled descriptor. The distances between all training compounds and the centroid were calculated, and the largest distance was adopted as the threshold d_t_.  Then, the Euclidean distance d_Euc,i_ between the test compound i and |

| **Aspect of the User Case/System to be Simulated** | | |
| --- | --- | --- |
|  |  | the centroid was calculated.  In case the d_Euc,i_ of compound i is larger than the d_t_, the compound is considered outside the AD.  The similarity-based AD was adopted for the fingerprint representation of compounds. This methodology computes the similarity matrix between the training compounds and the test compounds. If the average Tanimoto similarity between the test compound and its k most similar compounds in the training set exceeds the threshold S_T_, it is considered to be in the AD, otherwise the compound is considered outside the AD.  Software name for applicability domain assessment: Python v. 3.10 Limits of applicability domain:  Euclidean-based APD threshold (Mordred descriptors): 21.188 Similarity-based APD threshold (MACCS keys): 0.344  Similarity-based APD threshold (Morgan Fingerprints): 0.149 |
| **1.6** | **Publication on this data** | Pending publication |

| **The Data-based Model** | | | |
| --- | --- | --- | --- |
| **2.0** | **Equation type and name** |  | |
| **2.1** | **Database and type** |  | |
| **2.2** | **Equation** | Hypothesis |  |
|  |  | Physical quantities |  |

| **Computational detail of datamining operation** | | |
| --- | --- | --- |
| **3.1** | **Numerical Operations** |  |
| **3.2** | **Software tool** | Python v. 3.10 |
| **3.3** | **Margin Of Error** |  |

# **Section 9:** MODA for acute dermal toxicity model

Moda powered by [**Enalos Cloud Platform**](http://www.enaloscloud.novamechanics.com/)

MODA for

Simulated in project

**Acute Dermal Toxicity prediction**

| **OVERVIEW of the SIMULATION** | | | |
| --- | --- | --- | --- |
| **1** | **USER CASE** | Acute Dermal Toxicity prediction | |
| **2** | **CHAIN OF MODELS** | **Data Transformation 1** | Fingerprint-based model Data Transformation |
|  |  | **Data Transformation 2** | Fingerprint-based model Data Transformation |
|  |  | **Data Transformation 3** | Descriptor-based model Data Transformation |
|  |  | **Model 4** | Prediction model Data based Model |
|  |  | **Model 5** | Applicability Domain model Data based Model |
| **3** | **PUBLICATION PEER - REVIEWING THE DATA** | DOI provided: No | |
| **4** | **ACCESS CONDITIONS** | Access type: Free Owner of workflow: Workflow access link: | |
| **5** | **WORKFLOW AND ITS RATIONALE** |  | |

**Workflow picture**


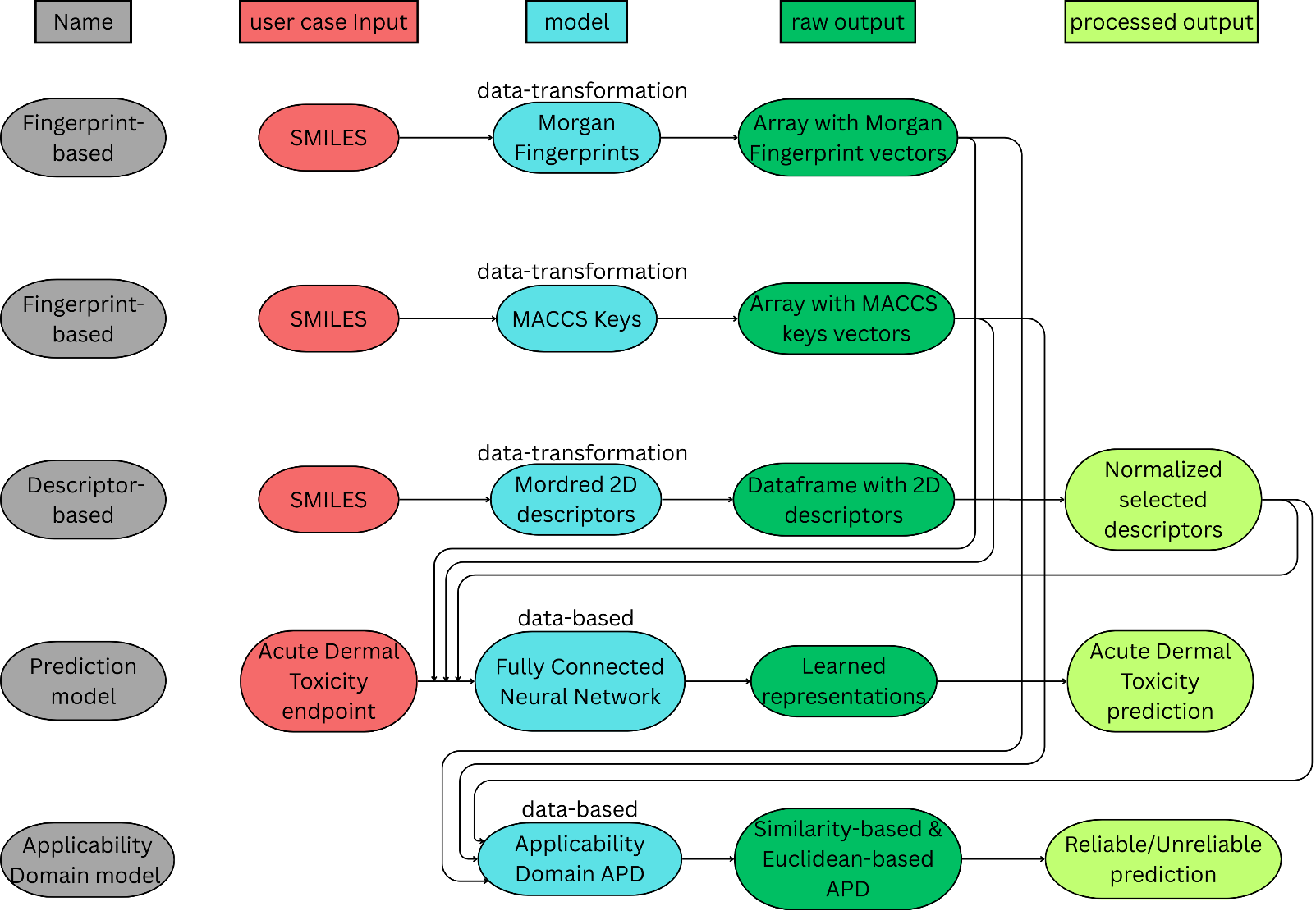


**Each physics-based model used in this simulation is to be documented in four chapters:**

1. Aspect of the User Case or system simulated with this model
2. Model: Please make sure the notions Physics Equation and Materials Relation are properly understood.
   1. Tightly coupled models can be written up collectively in one set of four tables. To solve tightly coupled PE one matrix is set up and solved in one go.
   2. For continuum models the PE is often the conservation equations coded up in bought software packages.
   3. Often the MR is established by the modeller.
3. Computational aspects include also a documentation of how the user case specifications are translated into computer language.
4. Post processing documents how the raw output of one simulation is processed into input for the next simulation. This information given under 4.1 in the first model will be the same as the "simulated input" information under 2.4 for the next model. This is the essence of model inter- operability!
5. Pre-processing before the first model can be depicted in pink as it is considered to be part of the user-case.

**Each data-based model in this simulation is to be documented in three chapters:**

1. Aspect of the User Case or system simulated with this data-based model
2. Data-based Model
3. Computational detail of the datamining operation

**Data Transformation 1**

Fingerprint-based model

| **Aspect of the User Case/System** | | |
| --- | --- | --- |
| **1.1** | **Aspect of the User Case to be simulated** | Convert SMILES to Morgan Fingerprints |
| **1.2** | **Material** | Small organic molecules |
| **1.3** | **Geometry** |  |
| **1.4** | **Time Lapse** |  |
| **1.5** | **Manufacturing process or in- service conditions** |  |
| **1.6** | **Publication on this data** | Pending publication |

| **Data Transformation** | | | |
| --- | --- | --- | --- |
| **2.0** | **Equation type and name** |  | |
| **2.1** | **Database and type** |  | |
| **2.2** | **Equation** | Hypothesis |  |
|  |  | Physical quantities |  |

| **Computational detail** | | |
| --- | --- | --- |
| **3.1** | **Numerical Operations** |  |
| **3.2** | **Software tool** | RDKit v. 2024.09.6 |
| **3.3** | **Margin Of Error** |  |

**Data Transformation 2**

Fingerprint-based model

| **Aspect of the User Case/System** | | |
| --- | --- | --- |
| **1.1** | **Aspect of the User Case to be simulated** | Convert SMILES to MACCS Keys |
| **1.2** | **Material** | Small organic molecules |
| **1.3** | **Geometry** |  |
| **1.4** | **Time Lapse** |  |
| **1.5** | **Manufacturing process or in- service conditions** |  |
| **1.6** | **Publication on this data** | Pending publication |

| **Data Transformation** | | | |
| --- | --- | --- | --- |
| **2.0** | **Equation type and name** |  | |
| **2.1** | **Database and type** |  | |
| **2.2** | **Equation** | Hypothesis |  |
|  |  | Physical quantities |  |

| **Computational detail** | | |
| --- | --- | --- |
| **3.1** | **Numerical Operations** |  |
| **3.2** | **Software tool** | RDKit v. 2024.09.6 |
| **3.3** | **Margin Of Error** |  |

**Data Transformation 3**

Descriptor-based model

| **Aspect of the User Case/System** | | |
| --- | --- | --- |
| **1.1** | **Aspect of the User Case to be simulated** | Convert SMILES to Mordred Descriptors |
| **1.2** | **Material** | Small organic molecules |
| **1.3** | **Geometry** |  |
| **1.4** | **Time Lapse** |  |
| **1.5** | **Manufacturing process or in- service conditions** |  |
| **1.6** | **Publication on this data** | Pending publication |

| **Data Transformation** | | | |
| --- | --- | --- | --- |
| **2.0** | **Equation type and name** |  | |
| **2.1** | **Database and type** |  | |
| **2.2** | **Equation** | Hypothesis |  |
|  |  | Physical quantities |  |

| **Computational detail** | | |
| --- | --- | --- |
| **3.1** | **Numerical Operations** |  |
| **3.2** | **Software tool** | Mordred v. 2.0.6 |
| **3.3** | **Margin Of Error** |  |

**Model 4**

Prediction model

| **Aspect of the User Case/System to be Simulated** | | |
| --- | --- | --- |
| **1.1** | **Aspect of the User Case to be simulated** | Acute dermal toxicity chemical records  Acute dermal toxicity is an adverse effect caused by a test chemical following a single uninterrupted expose by dermal application over a short period of time.  Endpoint Units: NA  Acute dermal toxicity data were collected from the Registration, Evaluation, Authorization and restriction of Chemicals (REACH) Study Results Database [1], the ToxValDB [2], and the literature [3].  [1] ECHA (European Chemical Agency), OECD (Organization for Economic Co- and operation and Development)., REACH Study Results - IUCLID, 2019. Accessed: Feb. 02, 2025. [Online]. Available: <https://iuclid6.echa.europa.eu/reach-study-results>  [2] R. Judson, “ToxValDB: Compiling Publicly Available In Vivo Toxicity Data,” 2019, The United States Environmental Protection Agency’s Center for Computational Toxicology and Exposure. doi: 10.23645/EPACOMPTOX.7800653  [3] S. Creton et al., “Acute toxicity testing of chemicals—Opportunities to avoid redundant testing and use alternative approaches,” Crit. Rev. Toxicol., vol. 40, no. 1, pp. 50–83, Jan. 2010, doi: 10.3109/10408440903401511  Complete dataset:  Toxics: 382  Non- toxics: 2234  Toxics / non- toxics: 0.17  Balanced dataset:  Toxics: 382  Non- toxics: 382  Toxics / non- toxics: 1.0 |

| **1.2** | **Material** | Small organic molecules |
| --- | --- | --- |
| **1.3** | **Geometry** |  |
| **1.4** | **Time Lapse** |  |
| **1.5** | **Manufacturing process or in- service conditions** |  |
| **1.6** | **Publication on this data** | Pending publication |

| **The Data-based Model** | | |
| --- | --- | --- |
| **2.0** | **Equation type and name** | Explicit algorithm:  Learning rate: 1e-3, Batch size: 32, Number of nodes of first dense  layer: 256, Number of nodes of second dense layer: 128, Dropout rate for the first dropout layer after the first dense layer: 0.2, Dropout rate for the second dropout layer after the second dense layer: 0.1  Availability of the datasets:  Available at the ChemPharos database: <https://db.chempharos.eu/datasets/Datasets.zul>  Available information for the datasets:   1. Chemical names (common names and/or IUPAC names) 2. CAS numbers 3. SMILES 4. InChI codes 5. Structural formula 6. MW   Pre-processing of data before modelling:  Checking SMILES structures with RDKit molecular object. Removal of columns containing NA values. In case of unbalanced dataset, an undersampling strategy was employed to result in a balanced dataset. Z-score normalization of descriptors. Variable selection of Mordred descriptors.  Availability of the external validation set:  Available at the ChemPharos database: <https://db.chempharos.eu/datasets/Datasets.zul>  Available information for the external validation set:   1. Chemical names (common names and/or IUPAC names) 2. CAS numbers 3. SMILES 4. InChI codes 5. Structural formula 6. MW   Other information about the external validation set:  77 molecules were included in the external validation (test set), which was not involved in model development, but it was rather used solely for validating purposes.  Experimental design of test set:  Randomly selected from the first partitioning of data (153 molecules). |

| **2.1** | **Database and type** |  | |
| --- | --- | --- | --- |
| **2.2** | **Equation** | Hypothesis | Fully connected neural network |
|  |  | Physical quantities | Descriptors in the model: The compounds were represented as Morgan fingerprints, MACCS keys, and Mordred descriptors.  Descriptor selection: The Morgan fingerprints and MACCS keys were used as bit vectors to represent the model. A combination of univariate and multivariate selection methods was then applied to pinpoint the most informative Mordred descriptors. First, an Analysis of Variance (ANOVA) was performed to identify descriptors with statistically significant differences between classes (e.g., toxics vs. non-toxics). Subsequently, L1-penalized linear regression (also known as Lasso regression) was used to further reduce descriptor complexity by imposing sparsity, effectively zeroing out less relevant coefficients and producing a concise set of Mordred descriptors.  Algorithm and descriptor generation: RDKit: We utilized RDKit Open-Source Toolkit for Cheminformatics for MACCS keys and Morgan Fingerprints generation [1]  [1] RDKit, RDKit: Open-source cheminformatics. Accessed: May 02, 2025. [Online]. Available: https://www.rdkit.org/  Mordred: We utilized Mordred package for molecular descriptor calculation. Mordred descriptors [2] constitute a set of more than 1,800 two- and three-dimensional descriptors that capture geometric, electronic, topological, and hybrid characteristics. In this study, only the two-dimensional Mordred descriptors were utilized to ensure consistency with other molecular representations.  [2] H. Moriwaki, Y.-S. Tian, N. Kawashita, and T. Takagi, “Mordred: a molecular descriptor calculator,” J. Cheminformatics, vol. 10, no. 1, p. 4, Dec. 2018, doi: 10.1186/s13321-018-0258-y  Software name and version for descriptor and algorithm generation:  RDKit v. 2024.09.6  Mordred v. 2.0.6  Chemicals/ Descriptors ratio: Our dataset contains 764 chemicals. The model uses a combined input feature space of 2278 descriptors, comprising of 2048-bit Morgan fingerprint vectors, 167-bit MACCS keys vectors and 62 Mordred descriptors. While the ratio of chemicals/descriptors should be high in classical QSPR modeling (due to risk of overfitting when the number of descriptors exceeds the number of chemicals), this is not a limiting factor in deep learning approaches. Fully Connected Neural Networks handle high-dimensional input spaces by learning complex representations. The number of hidden neurons in the second layer 128 which then pass through the output layer for the final classification of the compounds. |

| **Computational detail of datamining operation** | | |
| --- | --- | --- |
| **3.1** | **Numerical Operations** | Explicit Algorithm:  Learning rate: 1e-3 Batch size: 32  Number of nodes of first dense layer: 256  Number of nodes of second dense layer: 128  Dropout rate for the first dropout layer after the first dense layer: 0.2 Dropout rate for the second dropout layer after the second dense  layer: 0.1 |
| **3.2** | **Software tool** | Python v. 3.10 |
| **3.3** | **Margin Of Error** | Statistics for goodness-of-fit:  ACC: 0.997  AUC: 1.000  PRE: 0.993  SPE: 0.993  SEN: 1.000  MCC: 0.993  F1: 0.997  Robustness – Statistics obtained by 5-fold cross-validation (random  splitting of data):  ACC: 0.849  AUC: 0.923  PRE: 0.847  SPE: 0.846  SEN: 0.851  MCC: 0.698  F1: 0.849  Y-randomization:   \| Iteration \| ACC \| AUC \| SEN \| SPE \| MCC \| \| --- \| --- \| --- \| --- \| --- \| --- \| \| 1 \| 0.532 \| 0.551 \| 0.513 \| 0.553 \| 0.065 \| \| 2 \| 0.536 \| 0.523 \| 0.490 \| 0.584 \| 0.175 \| \| 3 \| 0.545 \| 0.561 \| 0.564 \| 0.526 \| 0.090 \| \| 4 \| 0.519 \| 0.536 \| 0.692 \| 0.342 \| 0.037 \| \| 5 \| 0.471 \| 0.518 \| 0.259 \| 0.689 \| 0.064 \|   Predictivity - Statistics obtained by external validation:  ACC: 0.792  AUC: 0.823  PRE: 0.848  SPE: 0.868  SEN: 0.718  MCC: 0.592  F1: 0.778 |

**Model 5**

Applicability Domain model

| **Aspect of the User Case/System to be Simulated** | | |
| --- | --- | --- |
| **1.1** | **Aspect of the User Case to be simulated** |  |
| **1.2** | **Material** |  |
| **1.3** | **Geometry** |  |
| **1.4** | **Time Lapse** |  |
| **1.5** | **Manufacturing process or in- service conditions** | In this study, two approaches were used: a Euclidean distance-based method for Mordred descriptor features and a Tanimoto similarity-based method for MACCS keys and Morgan fingerprints.  For Euclidean distance-based method, the Euclidean distance between the test compound and the centroid in training data is calculated and if this value is larger than the calculated APD threshold, the test compound is considered outside the applicability domain.  For similarity-based method, if the average Tanimoto similarity between the test compound and its k most similar compounds in the training exceed the APD threshold calculated on the training compounds, then is considered to be in the applicability domain.  For the models trained on concatenated features -combining Mordred descriptors and fingerprints- we considered both approaches independently to evaluate their individual contribution to the AD. Specifically, we applied the Euclidean distance-based approach to the descriptor subset and the similarity-based approach to the fingerprint subset, both MACCS and Morgan fingerprints. To determine whether a compound falls within the AD of the model, we adopted a consensus approach, considering a compound inside the AD if it meets at least two of the three defined thresholds.  For the Euclidean distance-based AD, the centroid of the training compounds was calculated as the mean of each scaled descriptor. The distances between all training compounds and the centroid were calculated, and the largest distance was adopted as the threshold d_t_.  Then, the Euclidean distance d_Euc,i_ between the test compound i and |

| **Aspect of the User Case/System to be Simulated** | | |
| --- | --- | --- |
|  |  | the centroid was calculated.  In case the d_Euc,i_ of compound i is larger than the d_t_, the compound is considered outside the AD.  The similarity-based AD was adopted for the fingerprint representation of compounds. This methodology computes the similarity matrix between the training compounds and the test compounds. If the average Tanimoto similarity between the test compound and its k most similar compounds in the training set exceeds the threshold S_T_, it is considered to be in the AD, otherwise the compound is considered outside the AD.  Software name for applicability domain assessment: Python v. 3.10 Limits of applicability domain:  Euclidean-based APD threshold (Mordred descriptors): 37.219 Similarity-based APD threshold (MACCS keys): 0.354  Similarity-based APD threshold (Morgan Fingerprints): 0.137 |
| **1.6** | **Publication on this data** | Pending publication |

| **The Data-based Model** | | | |
| --- | --- | --- | --- |
| **2.0** | **Equation type and name** |  | |
| **2.1** | **Database and type** |  | |
| **2.2** | **Equation** | Hypothesis |  |
|  |  | Physical quantities |  |

| **Computational detail of datamining operation** | | |
| --- | --- | --- |
| **3.1** | **Numerical Operations** |  |
| **3.2** | **Software tool** | Python v. 3.10 |
| **3.3** | **Margin Of Error** |  |
